# Novel mechanism of neuronal hypoxia response: HIF-1α/STOML2 mediated PINK1-dependent mitophagy activation against neuronal injury

**DOI:** 10.1038/s41420-026-02960-z

**Published:** 2026-02-21

**Authors:** Yuning Li, Zirui Xu, Zhengming Tian, Mengyuan Guo, Qianqian Shao, Yingxia Liu, Yakun Gu, Feiyang Jin, Xunming Ji, Jia Liu

**Affiliations:** 1 Beijing Institute of Brain Disorders, Laboratory of Brain Disorders, Hypoxia Conditioning Translational Laboratory of Clinical Medicine, Ministry of Science and Technology, Collaborative Innovation Center for Brain Disorders, Capital Medical University, Beijing, China; 2 Department of Neurosurgery, Xuanwu Hospital, Capital Medical University, Beijing, China; 3 Chinese Institutes for Medical Research, Beijing, China

**Keywords:** Cellular neuroscience, Mitophagy

## Abstract

Hypoxic stress contributes to brain disorders by causing neuronal injury, making it crucial to understand neuronal hypoxic response mechanisms for disease resistance. In the early stage of stress, neurons initiate a series of compensatory pathways to resist cell damage, but the underlying mechanisms have not been fully elucidated. In this study, we found that hypoxia transiently activates PTEN-induced kinase 1 (PINK1)-dependent mitophagy in the early stage before cell damage and neurological dysfunction. When PINK1-dependent mitophagy is inhibited, neuronal injury begins to exacerbate. Under hypoxia, overexpression of PINK1 can resist neuronal injury, while knockdown of PINK1 aggravates neuronal injury, revealing that PINK1-dependent mitophagy plays a key role in neuronal compensatory hypoxia response. Mechanistically, in the early stage of hypoxia, the nuclear translocation of HIF-1α increases, mediating the transcription of its downstream target molecule STOML2. STOML2 translocates to the outer mitochondrial membrane and participates in the cleavage of PGAM5. These processes initiate PINK1-dependent mitophagy. Knockdown of HIF-1α, STOML2, or PGAM5 inhibits mitophagy and worsens hypoxia-induced dysfunction, highlighting this pathway’s importance. Intermittent hypoxia, a conditioning strategy, stimulates endogenous protection. Notably, it activates the HIF-1α/STOML2 axis, inducing PINK1-dependent mitophagy and protecting neurons. In conclusion, our study reveals a new “self-protection” mechanism of neurons against hypoxic stress and discovers that intermittent hypoxia can effectively activate this pathway to resist neuronal injury, providing new insights into the mechanisms and interventions of hypoxia-related nerve injury.

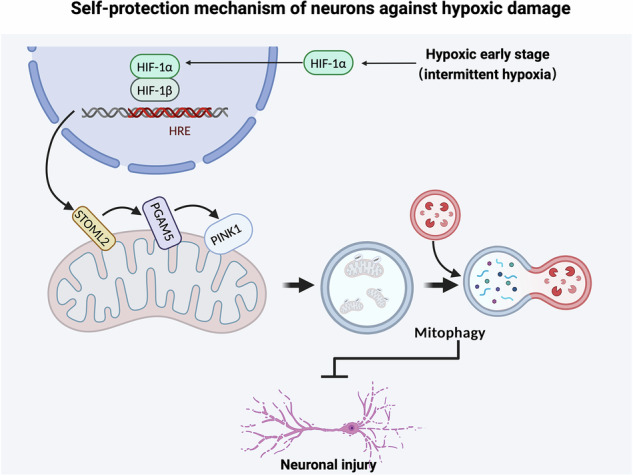

## Introduction

Hypoxia is implicated in a variety of central nervous system (CNS) disorders, such as stroke and Parkinson’s disease, suggesting that hypoxia may act as a co-factor in neurological injury [1]. One of the primary mechanisms by which hypoxia induces damage is through the disruption of mitochondrial function [2]. Mitochondria, the cell’s energy powerhouse, are responsible for oxidative phosphorylation, the process that produces ATP (adenosine triphosphate), the cell’s main energy currency [3]. The efficiency of this process is critical for maintaining cellular homeostasis, particularly in energy-demanding tissues like the brain [4, 5]. Under hypoxic conditions, mitochondrial dysfunction is commonly observed and has been implicated as a central pathogenic factor in several neurological disorders [6]. Dysfunctional mitochondria lead to the accumulation of reactive oxygen species (ROS), changes in mitochondrial dynamics, and eventually, cellular apoptosis [7, 8]. The accumulation of these damaged mitochondria contributes to neuronal injury in hypoxic environments. Therefore, understanding how hypoxia disrupts mitochondrial function and exploring the cellular mechanisms that mitigate this damage could reveal novel therapeutic targets for hypoxia-related neurological injuries.

One critical mechanism for mitigating mitochondrial dysfunction is mitophagy, a selective form of autophagy that plays a crucial role in maintaining mitochondrial quality control by targeting and degrading dysfunctional or damaged mitochondria [9]. Although macroautophagy has similar functions, mitophagy is more specific and effective in dealing with dysfunctional mitochondria [10]. Mitophagy is essential for the proper maintenance of cellular homeostasis, particularly in high-energy-demanding tissues such as neurons and muscles, where the proper functioning of mitochondria is critical for survival [11, 12]. Currently, a literature review summarizes the factors affecting mitophagy, among which hypoxia is an important way to activate mitophagy [13]. Studies have shown that FUNDC1 (FUN14 domain-containing protein 1) is the classical mitophagy receptor in response to hypoxia [14, 15]. However, after conducting more literature searches, we found that not only FUNDC1 but also a variety of other factors can mediate the activation of mitophagy under hypoxic stress. For example, PINK1 (PTEN-induced kinase 1), PINK1 is a classical mitophagy receptor [16]. Hypoxia has been shown to promote PINK1-dependent mitophagy [17]. Although it is known that hypoxia promotes PINK1 accumulation and mitophagy activation, the exact mechanisms remain unclear. Understanding these mechanisms could lead to the identification of novel therapeutic targets for treating diseases related to mitochondrial dysfunction under hypoxic stress.

In this study, we found that early-stage hypoxia activates PINK1-dependent mitophagy through the HIF-1α/STOML2/PGAM5 pathway, providing neuroprotection against hypoxic damage. To further investigate this, we performed knockdown experiments targeting these molecules. Additionally, we observed that intermittent hypoxia (IH) may also exert neuroprotective effects by activating this mitophagy pathway, offering promising clinical applications in the treatment of neurodegenerative diseases and conditions involving mitochondrial dysfunction.

## Results

### Mitophagy undergoes transient compensatory activation in the early hypoxic stage to resist cell damage

**Fig. 1:**
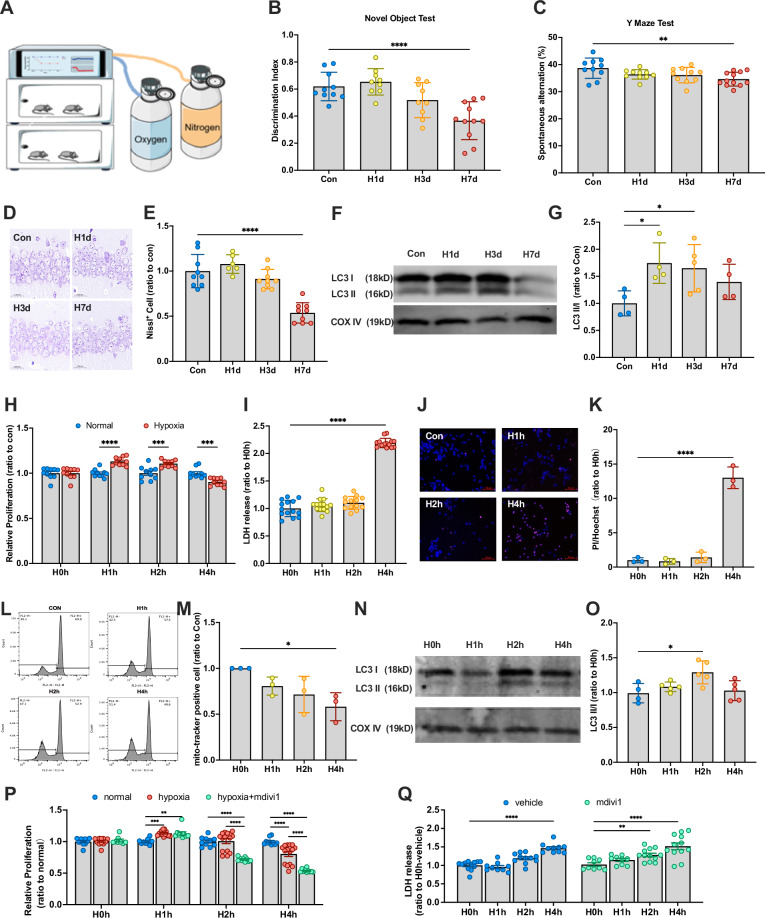
Neurons resist cellular damage by activating mitophagy in the early stage of hypoxia. **A** Specific hypoxia patterns. Mice were placed in hypoxic chambers with an O_2_ concentration of 13%. Constant levels of nitrogen and oxygen were maintained to achieve persistent hypoxia. **B**, **C** Mice were continuously treated with hypoxia for 0,1, 3, and 7 days (Con, H1d, H3d, H7d), and behavioral tests were performed at different time points. **B** Detection and statistical analysis of Novel Object Test in mice of each group. **C** Detection and statistical analysis of the Y Maze Test in mice of each group. **D** Nissl staining was used to evaluate the neuronal arrangement in the hippocampus. **E** The levels of Nissl-positive cells in mice of each group. **F** The levels of LC3 in the hippocampus: mitochondrial fraction were detected by Western blotting using COX IV as the internal reference. **G** Statistical analysis of LC3 II/I ratio in mice of each group. Statistical analysis of Western blot was performed by homogenization, setting the normoxia group value as 1.0. **H** SY5Y cells were cultured with 21% O_2_(normoxia) and 1% O_2_ (hypoxia) for 0, 1, 2 and 4 h, the cell relative proliferation was detected using the CCK-8 assay. **I** SY5Y cells were cultured with 1% O_2_ for 0, 1, 2, and 4 h, and the cytotoxicity was detected using the LDH assay. **J** The effect of hypoxic treatment on apoptosis was observed using PI/Hoechst staining kit and observed under a fluorescence microscope. **K** Statistical analysis of PI/Hoechst in different groups. **L** Following mitotracker staining, flow cytometry was performed to detect mitochondrial damage in SY5Y cells. **M** Statistical analysis of healthy mitochondrial counting. **N** The levels of LC3 in the mitochondrial fraction in SY5Y cells were detected by Western blotting using COX IV as the internal reference. **O** Statistical analysis of LC3 II/I ratio in mice of each group. Statistical analysis of Western blot was performed by homogenization, which means setting the value of the H 0 h group as 1. **P** Cell proliferation was evaluated using CCK-8 assay. Analysis was performed by homogenization, setting the normoxia group value as 1.0. **Q** The cytotoxicity was detected using LDH assay after suppressing mitophagy by mdivi1. Data were analyzed using ANOVA and post hoc Tukey’s tests. *p < 0.05, **p < 0.01, ***p < 0.001, ****p < 0.0001.

Adult C57BL mice were continuously exposed to 13% O₂, the oxygen concentration found on the Tibetan Plateau, for 0, 1, 3, and 7 days (Con, H1d, H3d, H7d). And we identify the H1d and H3d as early stages of hypoxia, while H7d as long-term hypoxic treatment. They subsequently underwent behavioral and postmortem histological analyses to assess neurological damage (Fig. 1A). To evaluate cognitive function, we performed the Novel Object Test, which evaluates novel object exploration and spatial exploration, respectively. Behavioral analysis revealed that while H1d and H3d mice maintained normal cognitive performance, H7d mice exhibited significant cognitive decline, indicating that prolonged hypoxia impairs cognitive function (Fig. 1B, C). Histological analysis of hippocampal neurons using Nissl staining revealed that neuronal arrangements in the Con, H1d, and H3d groups were orderly, whereas H7d mice exhibited disorganized hippocampal neuronal structures (Fig. 1D). Quantification of Nissl-positive cells, which represent viable neurons, further confirmed a significant decrease exclusively in the H7d group (Fig. 1E). These findings align with previous reports of cognitive impairment associated with prolonged hypoxia, suggesting that hypoxia-induced neuronal damage does not occur immediately. Instead, certain protective mechanisms may allow neurons to withstand early-stage hypoxic conditions. To investigate whether mitophagy contributes to hypoxia resistance, we first assessed LC3-I and LC3-II levels in mitochondrial fractions via Western blot analysis (Fig. 1F). The conversion of LC3-I to LC3-II serves as a marker of mitophagy activation. An increase in LC3-II/I ratio indicates mitophagy activation, which was significantly elevated in H1d and H3d groups (Fig. 1G). This suggests that mitophagy may play a crucial role in protecting against hypoxia-induced cognitive impairment. In summary, mitophagy is activated during the early stages of hypoxia (1–3 days) and may play a critical role in protecting against hypoxia-induced cognitive impairment.

To evaluate neuronal responses to hypoxia, we utilized the SH-SY5Y cell line and cultured the cells in 1% O₂. Cell viability was assessed using the CCK-8 assay, which revealed a significant increase in proliferation in the H1h and H2h groups, whereas a significant decline was observed in the H4h group (Fig. 1H). Cytotoxicity was assessed using the LDH assay, which showed a significant increase in LDH release exclusively in the H4h group (Fig. 1I). Apoptosis rates were evaluated using PI/Hoechst staining, with analysis revealing a marked increase in the PI/Hoechst ratio in the H4h group (Fig. 1J, K). These results are consistent with findings in mice, further supporting that hypoxia-induced neuronal damage occurs at a later phase of hypoxic exposure. To assess mitochondrial integrity, cells were stained with mito-tracker dye and analyzed by flow cytometry. The number of mito-tracker-positive (healthy) mitochondria was significantly reduced in the H4h group, indicating mitochondrial damage. To determine whether mitophagy protects against hypoxia-induced damage, we assessed LC3-II/I levels via Western blot analysis (Fig. 1N) and found a significant increase in LC3-II/I in the H2h group (Fig. 1O). Furthermore, to confirm the functional role of mitophagy, we inhibited mitophagy using mdivi-1. After mdivi-1 treatment, cell viability significantly decreased in the H2h group, suggesting that blocking mitophagy accelerates hypoxia-induced neuronal damage. Moreover, LDH release was significantly increased in H2h as well as H4h, further indicating that mitophagy is critical for neuronal survival under hypoxia. In summary, mitophagy is activated in neurons under hypoxic conditions and plays a protective role in preventing hypoxia-induced damage.

### PINK1-dependent mitophagy is an indispensable pathway for neurons to resist cell damage induced by hypoxia

**Fig. 2:**
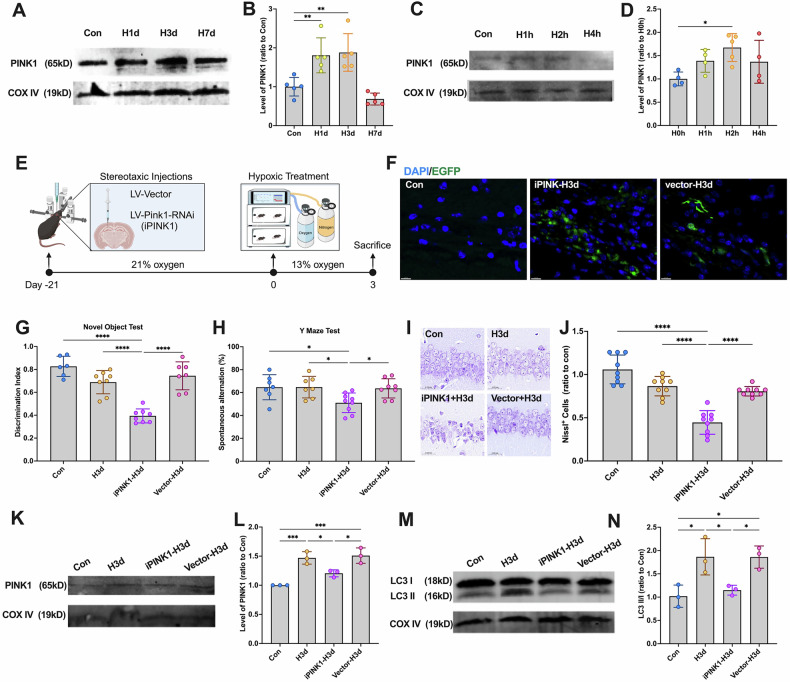
PINK1-dependent mitophagy mediates the resistance against hypoxic damage. **A** Mice were continuously treated with hypoxia for 0,1, 3, and 7 days (Con, H1d, H3d, H7d), and the levels of PINK1 in the hippocampus: mitochondrial fraction were detected by Western blotting using COX IV as the internal reference. **B** Statistical analysis of PINK1 in mice of each group. Statistical analysis of Western blot was performed by homogenization, setting the normoxia group value as 1.0. **C** SY5Y cells were continuously treated with hypoxia for 0,1, 2, and 4 h (H0h, H1h, H2h, H4h), and the levels of PINK1 in the neuron: mitochondrial fraction were detected by Western blotting using COX IV as the internal reference. **D** Statistical analysis of PINK1 in cells of each group. Statistical analysis of Western blot was performed by homogenization, setting the normoxia group value as 1.0. **E**–**N** Mice were administered the lentiviral targeting PINK1 and then received continuous hypoxic treatment for 3 days. Mice were grouped in 4 (Con, H3d, iPINK1-H3d and vector-H3d). **E** The timeline of lentiviral injection and hypoxic treatment. **F** Expression of LV-Pink1-RNAi-EGFP in the hippocampus was observed under a fluorescence microscope. **G** Detection and statistical analysis of Novel Object Test in mice of each group. **H** Detection and statistical analysis of the Y Maze Test in mice of each group. **I** Nissl staining was used to evaluate the neuronal arrangement in the hippocampus. **J** The levels of Nissl-positive cells in mice of each group. **K** The levels of PINK1 in mice of each group in the hippocampus: mitochondrial fraction were detected by Western blotting using COX IV as the internal reference. **L** Statistical analysis of PINK1 in mice of each group. Statistical analysis of Western blot was performed by homogenization, setting the normoxia group value as 1.0. **M** The levels of LC3 in the hippocampus: mitochondrial fraction were detected by Western blotting using COX IV as the internal reference. **N** Statistical analysis of LC3 II/I ratio in mice of each group. Data were analyzed using ANOVA and post hoc Tukey’s tests. *p < 0.05, **p < 0.01, ***p < 0.001, ****p < 0.0001.

To identify key mediators of hypoxia-induced mitophagy, we examined whether PINK1 (PTEN-induced kinase 1) regulates mitophagy activation. As a classical mitophagy receptor [16], PINK1 has been implicated in hypoxia-induced mitophagy, though the precise activation mechanism remains unclear. To address this, we isolated mitochondrial fractions and analyzed PINK1 levels. Western blot analysis revealed a significant increase in mitochondrial PINK1 expression in H1d and H3d mice (Fig. 2A, B) and H2h cells (Fig. 2C, D), confirming hypoxia-induced PINK1 upregulation. These findings suggest that PINK1-dependent mitophagy plays a crucial role in hypoxic adaptation.

To validate PINK1’s role, mice were administered lentiviral constructs targeting PINK1 and exposed to hypoxia for 3 days before undergoing behavioral and histological analysis (Fig. 2E). EGFP labeling confirmed successful viral transduction (Fig. 2F). As expected, iPINK1-H3d mice exhibited cognitive decline, while H3d and vector-H3d mice showed no significant impairment (Fig. 2G, H). Consistently, Nissl staining revealed significant neuronal damage in iPINK1-H3d mice, whereas H3d and vector-H3d groups maintained hippocampal integrity (Fig. 2I, J). These findings indicate that PINK1 protects against hypoxia-induced neuronal damage.

To confirm the role of PINK1 in mitophagy activation, we performed a Western blot analysis to assess its expression in mitochondrial fractions (Fig. 2K, L). The results showed that PINK1 was significantly upregulated in H3d but was reduced upon inhibition. Next, to determine whether PINK1 mediates hypoxia-induced mitophagy, we analyzed LC3 expression via Western blot (Fig. 2M). Results demonstrated that mitophagy activation, indicated by increased LC3-II/I ratios, was abolished in iPINK1-H3d mice, confirming that hypoxia-induced mitophagy is PINK1-dependent (Fig. 2N). Meanwhile, after injection of PINK1 overexpressing lentivirus, we found that the increase in mitophagy level would not be inhibited (Fig. S1), further suggesting that early hypoxia did activate PINK1-dependent mitophagy.

### Full-length PGAM5 mediates PINK1-dependent mitophagy under hypoxia

Early hypoxia activates PINK1-dependent mitophagy to mitigate neuronal injury. However, the mechanism by which PINK1 is stabilized on the outer mitochondrial membrane (OMM) and its functional role remains unclear.

**Fig. 3:**
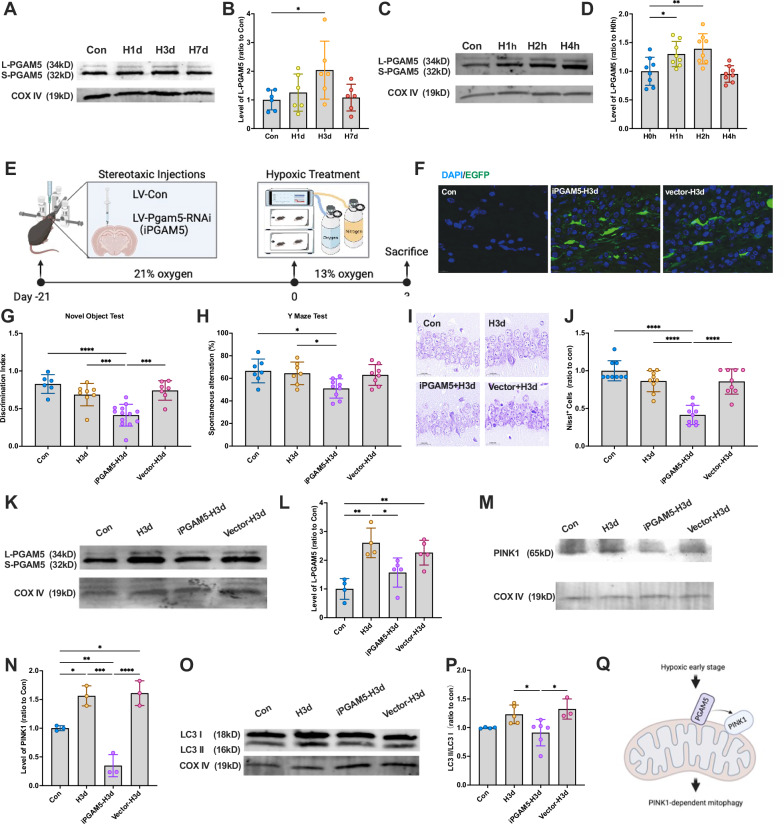
L-PGAM5 mediates PINK1-dependent mitophagy. **A** Mice were continuously treated with hypoxia for 0,1, 3, and 7 days (Con, H1d, H3d, H7d), and the levels of PGAM5 in the hippocampus mitochondrial protein were detected by Western blotting using COX IV as the internal reference. **B** Statistical analysis of L-PGAM5 in mice of each group. Statistical analysis of Western blot was performed by homogenization, setting the normoxia group value as 1.0. **C** SY5Y cells were continuously treated with hypoxia for 0,1, 2, and 4 h (H0h, H1h, H2h, H4h), and the levels of PGAM5 in the neuron mitochondrial protein were detected by western blotting using COX IV as the internal reference. **D** Statistical analysis of L-PGAM5 in cells of each group. Statistical analysis of western blot was performed by homogenization, setting the normoxia group value as 1.0. **E**–**N** Mice were administered the lentiviral targeting PGAM5 and then received continuous hypoxic treatment for 3 days. Mice were grouped in 4 (Con, H3d, iPGAM5-H3d and vector-H3d). **E** The timeline of lentiviral injection and hypoxic treatment. **F** Expression of LV-Pgam5-RNAi-EGFP in the hippocampus was observed under a fluorescence microscope. **G** Detection and statistical analysis of Novel Object Test in mice of each group. **H** Detection and statistical analysis of the Y Maze Test in mice of each group. **I** Nissl staining was used to evaluate the neuronal arrangement in the hippocampus. **J** The levels of Nissl-positive cells in mice of each group. **K** The levels of L-PGAM5 in mice of each group in the hippocampus: mitochondrial fraction were detected by Western blotting using COX IV as the internal reference. **L** Statistical analysis of L-PGAM5 in mice of each group. **MM)** The levels of PINK1 in mice of each group in the hippocampus: mitochondrial fraction were detected by Western blotting using COX IV as the internal reference. **N** Statistical analysis of PINK1 in mice of each group. **O** The levels of LC3 in the hippocampus: mitochondrial fraction were detected by Western blotting using COX IV as the internal reference. **P** Statistical analysis of LC3 II/I ratio in mice of each group. **Q** The mechanism diagram of the pathway. Data were analyzed using ANOVA and post hoc Tukey’s tests. *p < 0.05, **p < 0.01, ***p < 0.001, ****p < 0.0001.

Our early research showed that PGAM5 and STOML2 significantly increased after Hypoxia. These two factors are associated with mitophagy [18]. PGAM5 is a serine/threonine phosphatase on mitochondria [19], and previous studies suggest that full-length PGAM5 (L-PGAM5) facilitates PINK1 stabilization on the OMM [20, 21]. To explore the role of L-PGAM5 in early hypoxia-induced mitophagy, we isolated mitochondrial proteins from hippocampal tissues and SH-SY5Y cells for Western blot analysis. Results showed a significant increase in mitochondrial L-PGAM5 levels after 3 days of hypoxia in mice (Fig. 3A, B) and after 1–2 h of hypoxia in cells (Fig. 3C, D).

To determine whether PINK1 residency on mitochondria is necessary for mitophagy activation, we knocked down PGAM5 in the hippocampus via lentiviral injection (LV-Pgam5-RNAi) using stereotactic surgery and allowed three weeks for viral expression before hypoxia exposure (Fig. 3E). EGFP labeling confirmed successful viral transduction (Fig. 3F). As expected, iPGAM5-H3d mice exhibited cognitive decline, whereas H3d and vector-H3d mice showed no significant impairment (Fig. 3G, H). Nissl staining further revealed significant neuronal damage in iPGAM5-H3d mice, while hippocampal integrity was preserved in H3d and vector-H3d groups (Fig. 3I, J). These findings suggest that PGAM5 protects against hypoxia-induced neuronal damage.

Next, we performed Western blot analysis of mitochondrial proteins to confirm PGAM5 knockdown efficiency (Fig. 3K). L-PGAM5 levels were significantly reduced in the iPGAM5-H3d group, confirming successful knockdown (Fig. 3L). To investigate whether L-PGAM5 acts upstream of PINK1-dependent mitophagy, we examined mitochondrial PINK1 and LC3 levels in PGAM5-knockdown mice. PGAM5 depletion reversed the hypoxia-induced increase in mitochondrial PINK1 (Fig. 3M, N) and abolished mitophagy activation (Fig. 3O, P) in the early stages of hypoxia. These findings indicate that L-PGAM5 upregulation during early hypoxia is crucial for PINK1 stabilization and mitophagy activation (Fig. 3Q).

### Increased STOML2 on mitochondria upregulates full-length PGAM5 level

Our experimental results confirm that early hypoxia increases mitochondrial L-PGAM5, which activates PINK1-dependent mitophagy to protect against hypoxic injury. However, the mechanism by which L-PGAM5 is stabilized on mitochondria remains unclear. Previous studies suggest that STOML2, which increased significantly after hypoxia, is widely recognized for its role in mitochondrial biogenesis and inner membrane organization during cancer growing [22–25], such as gastric cancer. However, the role of STOML2 in the nervous system is currently unknown. STOML2 has been implicated in PGAM5 cleavage regulation [26], suggesting a potential upstream role in mitophagy activation.

**Fig. 4:**
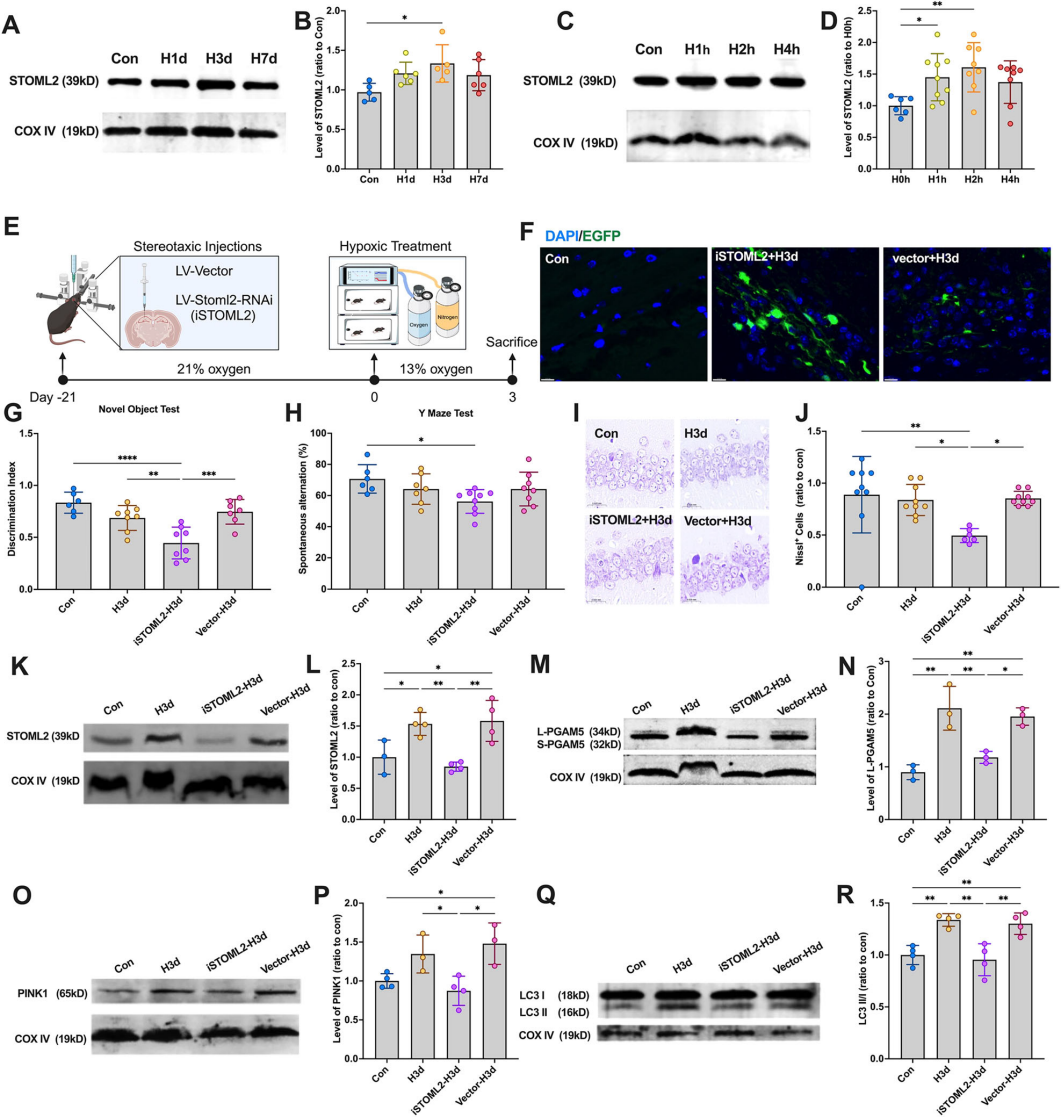
STOML2 helps stabilize L-PGAMS on the mitochondrial outer membrane. **A** Mice were continuously treated with hypoxia for 0,1, 3, and 7 days (Con, H1d, H3d, H7d), and the levels of STOML2 in the hippocampus: mitochondrial fraction were detected by Western blotting using COX IV as the internal reference. **B** Statistical analysis of STOML2 in mice of each group. Statistical analysis of Western blot was performed by homogenization, setting the normoxia group value as 1.0. **C** SY5Y cells were continuously treated with hypoxia for 0,1, 2, and 4 h (H0h, H1h, H2h, H4h), and the levels of STOML2 in the neuron: mitochondrial fraction were detected by Western blotting using COX IV as the internal reference. **D** Statistical analysis of STOML2 in cells of each group. Statistical analysis of Western blot was performed by homogenization, setting the normoxia group value as 1.0. **E**–**N** Mice were administered the lentiviral targeting STOML2 and then received continuous hypoxic treatment for 3 days. Mice were grouped in 4 (Con, H3d, iSTOML2-H3d and vector-H3d). **E** The timeline of lentiviral injection and hypoxic treatment. **F** Expression of LV-Stoml2-RNAi-EGFP in the hippocampus was observed under a fluorescence microscope. **G** Detection and statistical analysis of Novel Object Test in mice of each group. **H** Detection and statistical analysis of the Y Maze Test in mice of each group. **I** Nissl staining was used to evaluate the neuronal arrangement in the hippocampus. **J** The levels of Nissl-positive cells in mice of each group. **K** The levels of STOML2 in mice of each group in the hippocampus: mitochondrial fraction were detected by Western blotting using COX IV as the internal reference. **L** Statistical analysis of STOML2 in mice of each group. **M** The levels of L-PGAM5 in mice of each group in the hippocampus: mitochondrial fraction were detected by Western blotting using COX IV as the internal reference. **N** Statistical analysis of L-PGAM5 in mice of each group. **O** The levels of PINK1 in mice of each group in the hippocampus: mitochondrial fraction were detected by Western blotting using COX IV as the internal reference. **P** Statistical analysis of PINK1 in mice of each group. **Q** The levels of LC3 in the hippocampus: mitochondrial fraction were detected by Western blotting using COX IV as the internal reference. **R** Statistical analysis of LC3 II/I ratio in mice of each group. Data were analyzed using ANOVA and post hoc Tukey’s tests. *p < 0.05, **p < 0.01, ***p < 0.001, ****p < 0.0001.

To investigate STOML2’s role, we examined its mitochondrial levels under early hypoxia using Western blot. Results showed a significant increase in mitochondrial STOML2 in H3d mice (Fig. 4A, B) and in SH-SY5Y cells after 2 h of hypoxia (H2h) (Fig. 4C, D), suggesting that STOML2 upregulation accompanies L-PGAM5-mediated activation of PINK1-dependent mitophagy.

To determine whether STOML2 stabilizes L-PGAM5 on mitochondria, we knocked down STOML2 in the hippocampus (LV-Stoml2-RNAi) via stereotactic injection and allowed three weeks for viral expression before hypoxia exposure (Fig. 4E). EGFP labeling confirmed successful transduction (Fig. 4F). As expected, iSTOML2-H3d mice exhibited cognitive decline, whereas H3d and vector-H3d mice showed no significant impairment (Fig. 4G, H). Nissl staining further revealed significant neuronal damage in iSTOML2-H3d mice, while hippocampal integrity was maintained in H3d and vector-H3d groups (Fig. 4I, J). These findings suggest that STOML2 protects against hypoxia-induced neuronal damage.

To confirm the functional role of STOML2 in mitophagy regulation, we knocked down STOML2 and assessed its mitochondrial protein levels via Western blot (Fig. 4K, L). Western blot analysis confirmed that STOML2 was successfully depleted in iSTOML2-H3d mice. Next, we examined whether STOML2 is necessary for L-PGAM5 stabilization by analyzing mitochondrial protein fractions (Fig. 4M, N). Meanwhile, our results showed that the knockdown of PGAM5 did not affect STOML2 expression (Fig. S2). The results showed that STOML2 knockdown reversed the hypoxia-induced increase in L-PGAM5 expression, indicating that STOML2 functions upstream of PGAM5 in mitophagy activation.

Next, we examined whether STOML2 upregulation enhances PINK1-dependent mitophagy. Western blot analysis revealed that STOML2 knockdown abolished hypoxia-induced PINK1 accumulation on mitochondria (Fig. 4O, P) and prevented mitophagy activation (Fig. 4Q, R). Together, these findings demonstrate that hypoxia-induced STOML2 upregulation stabilizes L-PGAM5, which in turn activates PINK1-dependent mitophagy to confer resistance against hypoxic injury. STOML2 is widely recognized for its role in mitochondrial biogenesis and inner membrane organization during cancer growth [21, 22]. However, our study uncovers a previously unknown function of STOML2 in regulating mitophagy under hypoxia.

### Hypoxia promotes STOML2 transcription through HIF-1α pathway and thus activates mitophagy

**Fig. 5:**
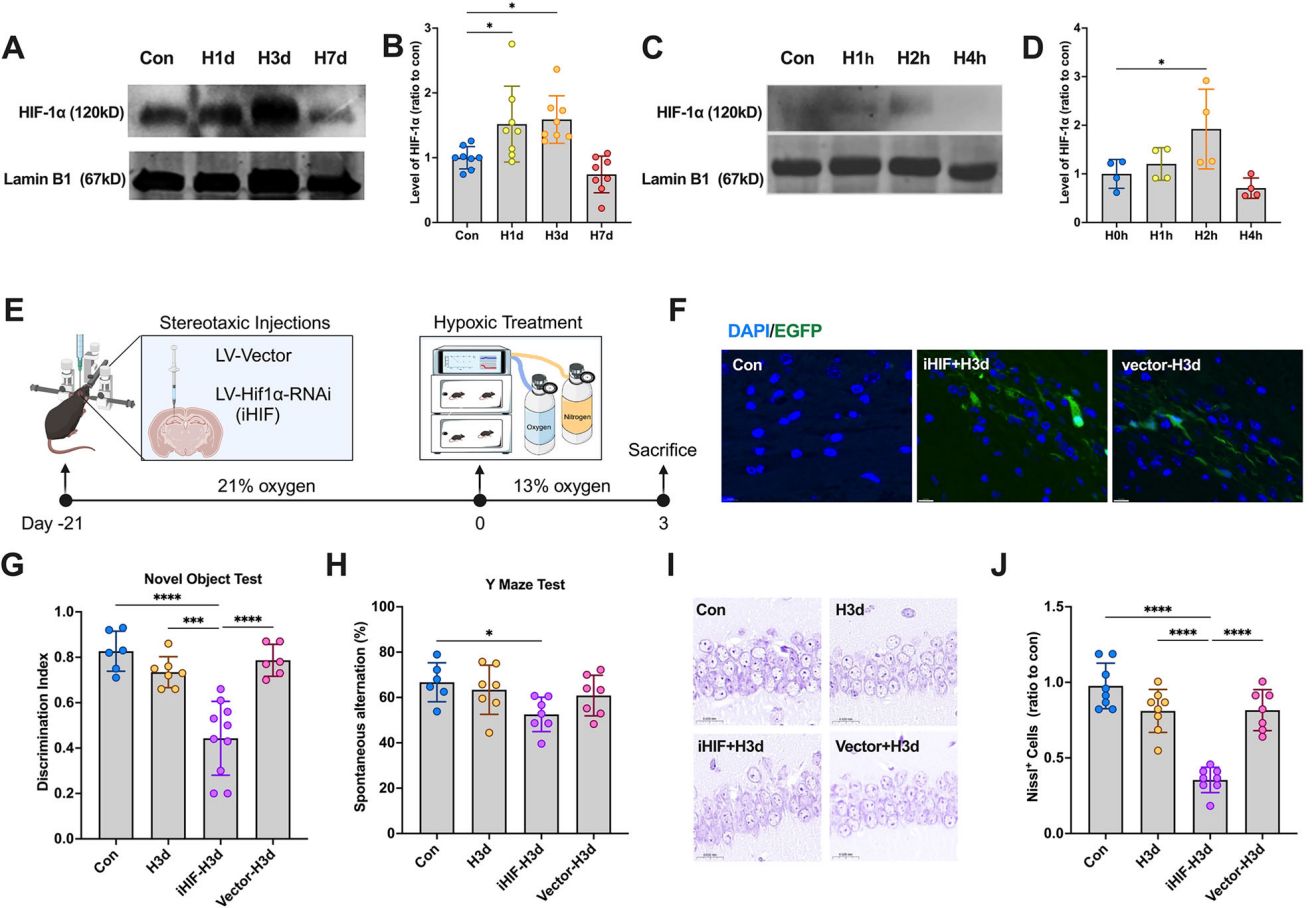
Neurons resist hypoxic damage by activating HIF-1α. **A** Mice were continuously treated with hypoxia for 0,1, 3, and 7 days (Con, H1d, H3d, H7d), and the levels of HIF1α in the hippocampus nucleus protein were detected by Western blotting using LaminB1 as the internal reference. **B** Statistical analysis of HIF-1α in mice of each group. Statistical analysis of Western blot was performed by homogenization, setting the normoxia group value as 1.0. **C** SY5Y cells were continuously treated with hypoxia for 0,1, 2, and 4 h (H0h, H1h, H2h, H4h), and the levels of HIF-1α in the neuron: mitochondrial fraction were detected by Western blotting using COX IV as the internal reference. **D** Statistical analysis of HIF-1α in cells of each group. Statistical analysis of Western blot was performed by homogenization, setting the normoxia group value as 1.0. **E**–**J** Mice were administered the lentiviral targeting HIF-1α and then received continuous hypoxic treatment for 3 days. Mice were grouped in 4 (Con, H3d, iHIF1α-H3d and vector-H3d). **E** The timeline of lentiviral injection and hypoxic treatment. **F** Expression of LV- Hif1α-RNAi-EGFP in the hippocampus was observed under a fluorescence microscope. **G** Detection and statistical analysis of Novel Object Test in mice of each group. **H** Detection and statistical analysis of the Y Maze Test in mice of each group. **I** Nissl staining was used to evaluate the neuronal arrangement in the hippocampus. **J** The levels of Nissl-positive cells in mice of each group. Data were analyzed using ANOVA and post hoc Tukey’s tests. *p < 0.05, **p < 0.01, ***p < 0.001, ****p < 0.0001.

To investigate HIF-1α nuclear translocation under hypoxia, we analyzed HIF-1α levels in nuclear fractions from mouse hippocampal tissues and SH-SY5Y cells using Western blot. Results showed a significant increase in nuclear HIF-1α in H1d and H3d mice (Fig. 5A, B) and in H2h cells (Fig. 5C, D), indicating that early hypoxia promotes HIF-1α nuclear entry. This suggests that hypoxia-induced mitophagy may be HIF-1α-dependent.

To confirm HIF-1α’s role, we knocked down HIF-1α in the hippocampus (LV-Hif1α-RNAi) via stereotactic injection and allowed three weeks for viral expression before hypoxia exposure (Fig. 5E). EGFP labeling confirmed successful transduction (Fig. 5F). As expected, iHIF-H3d mice exhibited cognitive decline, while H3d and vector-H3d mice showed no significant impairment (Fig. 5G, H). Nissl staining further revealed significant neuronal damage in iHIF-H3d mice, whereas hippocampal integrity was maintained in H3d and vector-H3d groups (Fig. 5I, J). These findings indicate that HIF-1α protects against hypoxia-induced neuronal damage.

**Fig. 6:**
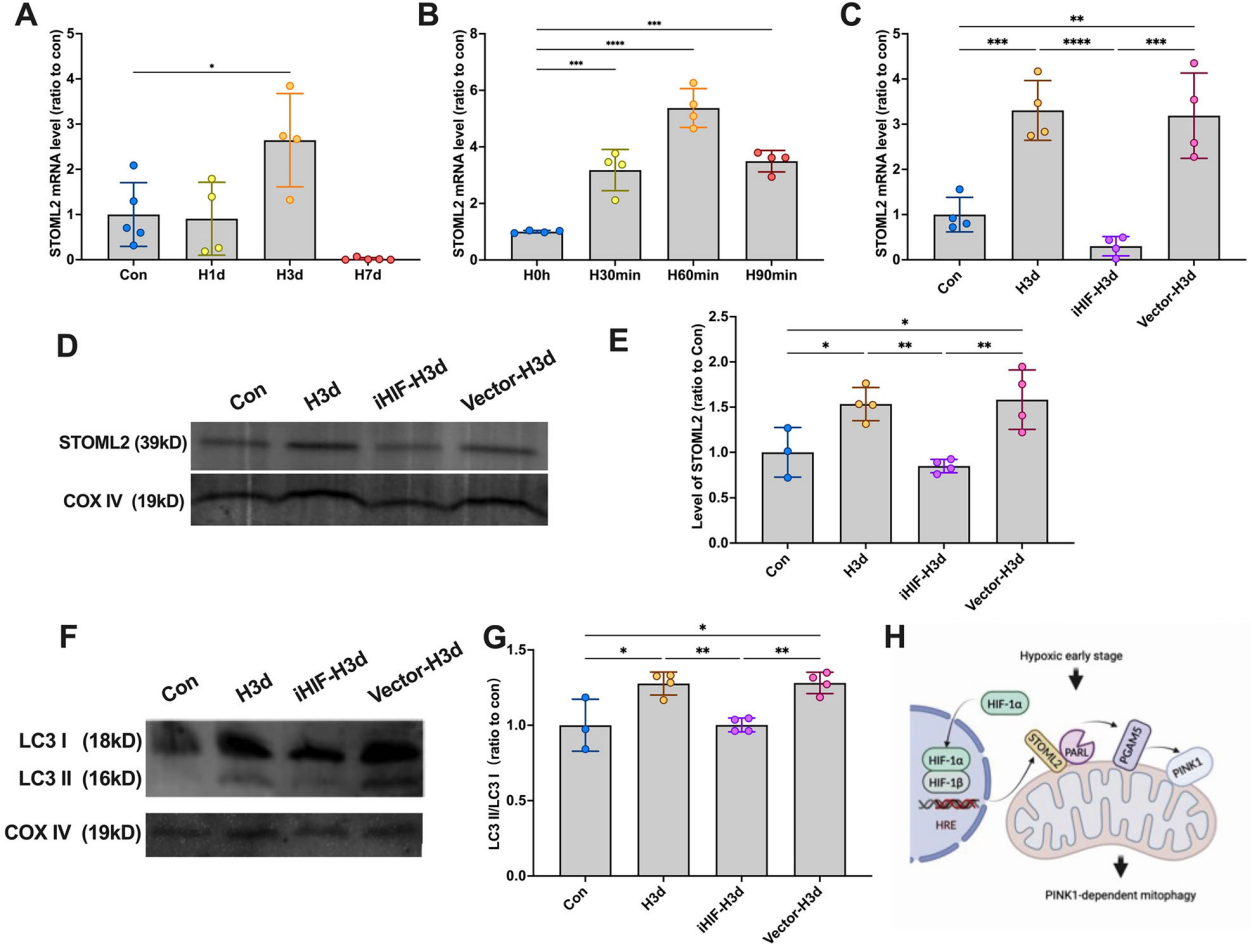
The STOML2/PGAMS/PINK1 signaling pathway is dependent on HIF-1α activation. **A** The levels of STOML2 mRNA were detected by qPCR in mice of each group. **B** The levels of STOML2 mRNA were detected by qPCR in cells of each group. **C**–**G** Mice were administered the lentiviral targeting HIF-1α and then received continuous hypoxic treatment for 3 days. Mice were grouped in 4 (Con, H3d, iHIF1α-H3d and vector-H3d). **C** The levels of STOML2 mRNA were detected by qPCR in mice of each group. **D** The levels of STOML2 in mice of each group in the hippocampus: mitochondrial fraction were detected by Western blotting using COX IV as the internal reference. **E** Statistical analysis of STOML2 in mice of each group. **F** The levels of LC3 in the hippocampus: mitochondrial fraction were detected by Western blotting using COX IV as the internal reference. **G** Statistical analysis of LC3 II/I ratio in mice of each group. **H** The mechanism diagram of the pathway. Data were analyzed using ANOVA and post hoc Tukey’s tests. *p < 0.05, **p < 0.01, ***p < 0.001, ****p < 0.0001.

STOML2 has been identified as a novel downstream target of HIF-1α [5]. To assess STOML2 transcriptional regulation by HIF-1α, we measured STOML2 mRNA levels via qPCR. In both mice (H3d) and SH-SY5Y cells (H30min, H60min, H90min), STOML2 mRNA was significantly increased, peaking at 60 min of hypoxia (Fig. 6A, B). Notably, HIF-1α knockdown abolished this upregulation (Fig. 6C), confirming that HIF-1α nuclear translocation enhances STOML2 transcription.

Western blot further verified successful HIF-1α knockdown, as nuclear HIF-1α levels were no longer elevated in iHIF-H3d mice (Fig. 6D, E). To determine whether HIF-1α acts upstream of mitophagy, we assessed mitochondrial LC3 levels following HIF-1α depletion. Results showed that HIF-1α knockdown abolished mitophagy activation in early hypoxia (Fig. 6F, G).

Collectively, our findings demonstrate that early hypoxia promotes HIF-1α nuclear translocation, which in turn upregulates STOML2 expression. This leads to enhanced L-PGAM5 stability and subsequent activation of PINK1-dependent mitophagy. We propose that the HIF-1α/STOML2/PGAM5/PINK1 axis represents a novel hypoxia-responsive signaling pathway that plays a crucial role in neuronal protection against hypoxic injury (Fig. 6H).

### Intermittent hypoxia activates PINK1-dependent mitophagy through the HIF-1α/STOML pathway to resist nerve damage

**Fig. 7:**
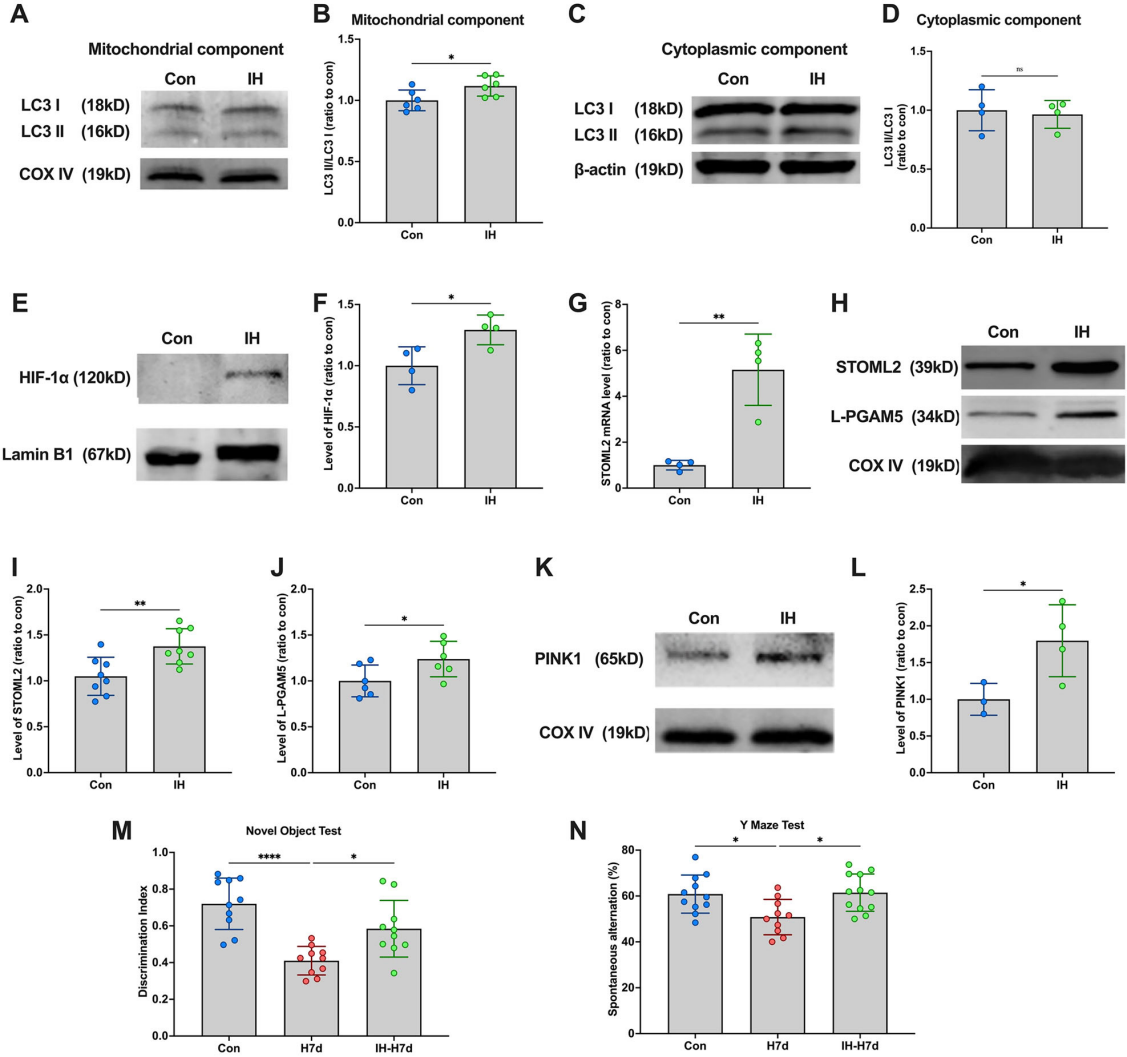
Intermittent hypoxic treatment activates PINK1-dependent mitophagy via the HIF-1α/STOML2 signaling pathways. **A** The levels of LC3 in the hippocampus: mitochondrial fraction were detected by Western blotting using COX IV as the internal reference. **B** Statistical analysis of LC3 II/I ratio in mice of each group. **C**, **D** The levels of LC3 in the hippocampus: cytoplasmic fraction were detected by Western blotting using β-actin as the internal reference. **G** Statistical analysis of LC3 II/I ratio in mice of each group. **E** The levels of HIF-1α in the hippocampus: nucleus fraction were detected by Western blotting using LaminB1 as the internal reference. **F** Statistical analysis of HIF-1α in mice of each group. **G** The levels of STOML2 mRNA were detected by qPCR in mice of each group. **H** The levels of STOML2 and L-PGAM5 in mice of each group in the hippocampus: mitochondrial fraction were detected by Western blotting using COX IV as the internal reference. **I** Statistical analysis of STOML2 in mice of each group. **J** Statistical analysis of L-PGAM5 in mice of each group. **K** The levels of PINK1 in mice of each group in the hippocampus: mitochondrial fraction were detected by Western blotting using COX IV as the internal reference. **L** Statistical analysis of PINK1 in mice of each group. **M**, **N** Mice received hypoxic treatment for 7 days after receiving intermittent hypoxic treatment and behavioral tests were performed. Mice were grouped into 3 (Con, H7d, IH+H7d). **M** Detection and statistical analysis of Novel Object Test in mice of each group. **N** Detection and statistical analysis of the Y Maze Test in mice of each group. Data was analyzed via paired two-tailed Student’s *t* test. *p < 0.05, **p < 0.01, ****p < 0.0001.

Previous studies suggest that intermittent hypoxia preconditioning (IH) can mitigate cognitive impairment in mice induced by chronic hypoxia. To investigate the underlying protective mechanisms, we examined whether mitophagy contributes to this effect. To assess mitophagy activation, we isolated mitochondrial and cytoplasmic proteins and performed Western blot analysis. Results showed a significant increase in LC3-II levels in mitochondria (Fig. 7A, B), whereas no such increase was observed in the cytoplasmic fraction (Fig. 7C, D). This indicates that IH triggers mitophagy through the intrinsic pathway during early hypoxia.

To determine whether IH activates the HIF-1α/STOML2/PGAM5/PINK1 pathway, we analyzed HIF-1α levels in nuclear fractions and found a significant increase after IH treatment (Fig. 7E, F). Further Western blot analysis of mitochondrial proteins revealed elevated STOML2, L-PGAM5, and PINK1 levels after IH (Fig. 7G, H, J–L). Additionally, qPCR analysis confirmed a significant increase in STOML2 mRNA following IH (Fig. 7I). These findings suggest that IH activates the HIF-1α/STOML2/PGAM5/PINK1 signaling pathway.

To evaluate whether this pathway contributes to cognitive protection, mice underwent behavioral testing after 7 days of persistent hypoxia following IH treatment. Results from the novel object recognition and Y-maze tests demonstrated that IH significantly alleviated cognitive impairment induced by chronic hypoxia (Fig. 7M, N).

These findings suggest that IH activates the HIF-1α/STOML2/PGAM5/PINK1 pathway, which in turn induces mitophagy and protects against hypoxia-induced cognitive decline. This pathway may serve as a potential neuroprotective mechanism in hypoxic conditions.

## Discussion

**Fig. 8:**
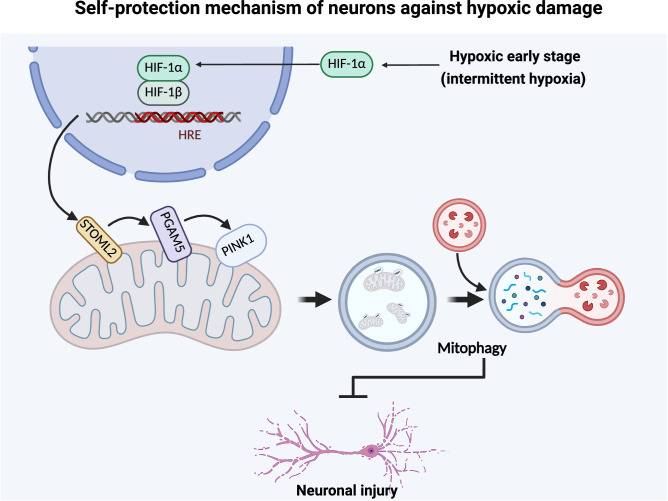
A novel neuronal hypoxia response mechanism. In the early stage of hypoxia or intermittent hypoxia treatment, PINK1-dependent mitophagy is activated by HIF-1α/STOML2-signaling to protect against hypoxia-induced neuronal injury.

Our findings demonstrate that PINK1-dependent mitophagy serves as a critical protective mechanism in early hypoxia, enabling neurons to selectively eliminate damaged mitochondria and maintain cellular homeostasis (Fig. 8). This process is initiated by the stabilization of HIF-1α, which upregulates STOML2 expression, leading to the stabilization of PGAM5 and ultimately activating PINK1-mediated mitophagy. Moreover, this study lays the theoretical foundation for exploring IH as a potential clinical intervention to enhance neuroprotection against hypoxic injury.

During early-stage hypoxia, mitophagy is transiently activated to remove dysfunctional mitochondria, allowing cells to adapt to reduced oxygen levels. However, prolonged hypoxia alters mitochondrial dynamics and cellular stress responses, causing mitophagy to shift from a protective adaptation to a maladaptive process. If mitophagy is sustained for too long, excessive mitochondrial clearance can lead to bioenergetic failure, cellular dysfunction, and even apoptosis. Previous studies have shown that the phosphorylation of FUNDC1 decreases, resulting in diminished mitophagy activity [27]. Similarly, studies in neurons have demonstrated that BNIP3/NIX-mediated mitophagy is transiently activated but suppressed as cells enter hypoxia-induced apoptosis [28]. These findings highlight the need for a tightly regulated mitophagy response, balancing mitochondrial clearance with cellular survival. In this study, we confirm that the HIF-1α/STOML2/PGAM5/PINK1 pathway functions as a novel and effective mechanism for mitophagy activation under hypoxia, as evidenced by knockdown experiments targeting key molecules in the pathway.

Studies have indicated that stabilized HIF-1α enhances mitophagy, reinforcing its role in maintaining mitochondrial homeostasis [29]. Our findings confirm that hypoxia-induced mitophagy is HIF-1α-dependent, underscoring the importance of this regulatory axis. Notably, HIF-1α activation is transient [30]—it peaks within 4–8 h and declines after 12–24 h in HeLa cells [31]. HIF-1α provides short-term neuroprotection by promoting blood vessel formation (VEGF) and metabolic adaptation [32], but prolonged HIF-1α activation can lead to blood-brain barrier disruption and edema [33]. This transient pattern supports our conclusion that mitophagy is similarly short-lived during early hypoxia, with HIF-1α playing a key role in the activation of PINK1-dependent mitophagy.

Given that STOML2 functions as a downstream target of HIF-1α [34], we propose a model in which early hypoxia stabilizes HIF-1α, upregulating STOML2 expression, which in turn maintains PGAM5 stability and activates PINK1-dependent mitophagy. Without PGAM5 stabilization, PINK1 is rapidly degraded, leading to impaired mitophagy and exacerbated neuronal injury under hypoxia [35]. Interestingly, previous studies have also shown that PGAM5 regulates mitochondrial fission by modulating DRP1 phosphorylation [36], indicating that its function in mitophagy may extend beyond PINK1 stabilization. Future research should explore whether PGAM5 influences mitochondrial dynamics, particularly the balance between mitophagy and mitochondrial fission-fusion regulation in response to hypoxic stress.

FUNDC1 is the canonical pathway that increases mitophagy after hypoxia. However, in our model, this process relies on PINK1. Unlike FUNDC1, which directly interacts with LC3 [37], PINK1 recruits Parkin, an E3 ubiquitin ligase, which ubiquitinates mitochondrial proteins to initiate mitophagy [38]. PGAM5 is involved in both pathways but plays distinct roles. PGAM5 functions as a phosphatase, dephosphorylating FUNDC1 to enhance its interaction with LC3 under hypoxia [39]. In contrast, PGAM5 stabilizes PINK1, preventing its degradation and facilitating mitophagy activation. Both pathways are crucial for maintaining mitochondrial homeostasis during hypoxia but operate through different mechanisms. Moreover, PINK1-dependent mitophagy is not exclusively triggered by sustained hypoxia, but can also be activated by various physiological or preconditioning stimuli, underscoring that its neuroprotective role extends well beyond canonical hypoxic responses.

Recent studies highlight the critical role of mitophagy in protecting against neurodegenerative pathology and cognitive impairment. Enhancing mitophagy reduces toxic protein burden, preserves synaptic integrity, and mitigates neuronal functional decline in models of AD and PD [40–45]. In parallel, activation of broader autophagy pathways has been shown to restore mitochondrial homeostasis and improve cognitive resilience following cerebral ischemia or metabolic stress, as well as in models of Alzheimer’s and Parkinson’s disease [46–49], underscoring the importance of maintaining efficient mitochondrial quality control. These findings support our conclusion that mitophagy is essential for sustaining neuronal function during hypoxia. In this context, the HIF-1α–STOML2–PGAM5–PINK1 axis identified here provides mechanistic insight into early hypoxic adaptation and represents a promising therapeutic target to prevent hypoxia-induced cognitive deficits.

In this study, we observed that after IH preconditioning, no cognitive decline was observed in mice following 7 days of continuous hypoxia. This neuroprotective effect was associated with the activation of the HIF-1α/STOML2/PGAM5 pathway, which subsequently enhanced PINK1-dependent mitophagy, promoting mitochondrial quality control and neuronal survival. Previous studies have shown that IH confers neuroprotection through multiple mechanisms: In models of cerebral ischemia, IH promotes mitochondrial biogenesis and prevents oxidative stress-induced apoptosis [50]; studies in stroke models have demonstrated that IH increases VEGF expression, improving cerebral blood flow and neuronal survival [51]; IH preconditioning suppresses pro-inflammatory cytokines (TNF-α, IL-6) and enhances antioxidant enzyme activity [52], thereby protecting neurons from hypoxia-induced damage. Notably, this study provides the first evidence demonstrating that IH specifically activates the HIF-1α/STOML2/PGAM5 axis, revealing a novel regulatory mechanism linking IH to enhanced mitophagy and neuroprotection.

A key limitation is that our experiments included only male mice; potential sex differences in cerebrovascular and cognitive responses to hypoxia were not assessed. Prior studies report that females often exhibit greater resistance to hypoxia-induced neuronal injury and maintain superior cerebrovascular reactivity, attributable in part to estrogen-mediated mitochondrial protection [53, 54]. Age is also likely to modulate oxygen-dependent regulation of neuronal metabolism and cognition, as aging exacerbates mitochondrial dysfunction and attenuates adaptive mitophagy [55, 56]. Accordingly, future work will include both sexes and multiple age cohorts to delineate how intermittent hypoxia (IH)–induced mitophagy contributes to neuroprotection across physiological contexts and to enhance the translational relevance of our findings.

In conclusion, we propose that the stabilization of HIF-1α initiates a novel process that enhances STOML2 expression, promotes PGAM5 stability, and ultimately activates PINK1-dependent mitophagy. This pathway represents a potential new target for treating hypoxia-related diseases. Furthermore, IH may mimic the early stages of hypoxia and serve as an exogenous activator of the HIF-1α/STOML2/PGAM5/PINK1 pathway, providing neuroprotection. Our findings also offer experimental support for the clinical application of IH, although further studies are needed to confirm its therapeutic potential.

## Materials and methods

### Animals

Adult male C57BL mice were purchased from SPF Biotechnology (Beijing, China). All animals were housed at room temperature under a 12/12 h light/dark cycle and had free access to food and water. All animal experiments were approved by the Animal Care and Use Committee of the Institute of Animal Management, Capital Medical University (permit no. AEEI-2022-073), and conducted in accordance with ethical requirements and ARRIVE guidelines.

### Hypoxic treatment

All mice were randomly assigned to the control group and each model group. Hypoxic mice were administered hypoxic treatment in a closed hypoxic chamber (China Innovation Instrument Co., Ltd, Ningbo, Zhejiang, China), which accurately set the desired hypoxic concentration and pattern. For chronic hypoxia, mice were treated continuously with 13% O2 for 1, 3, and 7 days. The hypoxic chamber was opened briefly for food and water additions every 3 days. Intermittent hypoxic mice were treated with 10 cycles of 5-min 13% O2 (hypoxia) and 5-min 21% O2 (normoxia) per day for 14 days. The IH-H7d group was followed by an additional continuous 3-day hypoxic treatment after IH treatment.

### Lentivirus Treatment

To investigate the knockdown effects of four target molecules (HIF-1α, STOML2, PGAM5, and PINK1), along with a negative control (vector), and the control vectors are scrambled shRNA sequences, five lentivirus treatment groups were established, with 10 animals per group: H3d-vector group: Experimental animals were injected with lentivirus via stereotactic injection and subjected to continuous hypoxia (13% O₂) for 3 days. H3d-iHIF group: Lentivirus was administered via stereotactic injection to specifically knock down HIF-1α, followed by 3 days of continuous hypoxia (13% O₂). H3d-iSTOML2 group: Lentivirus was injected to selectively knock down STOML2, followed by 3 days of continuous hypoxia (13% O₂). H3d-iPINK1 group: Lentivirus was injected to specifically knock down PINK1, followed by 3 days of continuous hypoxia (13% O₂). And the sequences of the shRNA lentiviruses employed, as well as other detailed information, are provided in the table below.

### Behavioral tests

The cognitive function of mice in each group was assessed using novel object recognition and Y-maze tests (Table 1). For the novel object recognition test, a 40 cm × 40 cm × 40 cm lidless rectangular box was used, with a camera positioned overhead. The experiment consisted of three phases: adaptation, familiarity, and testing. In the adaptation phase, each mouse was placed in the apparatus and allowed to explore freely for 5 min to acclimate. In the familiarity phase, two identical objects A (old object) were introduced, and mice were given 5 min to explore. In the testing phase, one object A was replaced with a novel object (differing in color and shape), and mice were allowed another 5 min of exploration. The time spent interacting with both objects was recorded. The discrimination index was calculated as (Time exploring new object−Time exploring old object)/(Time exploring new + old objects). Mice with baseline cognitive impairments were excluded from behavioral tests. All video recordings were analyzed blindly by researchers not involved in conducting the experiments.

**Table 1:** The sequences of the shRNA lentiviruses.

Construct Name	Target	Sequence
LV-HIF-1a-RNAi	HIF-1a	CCCATTCCTCATCCGTCAAAT
LV-Stoml2-RNAi	Stoml2	GAGTCCCTGAATGCCAACATT
LV-Pgam5-RNAi	Pgam5	AGAAGACGAGTTGACATCC
LV-Pink1-RNAi	Pink1	GCTGCAAATGTGCTGCACTTA
scramble	scramble	TTCTCCGAACGTGTCACGT

The Y maze is typically made of opaque material and shaped like the letter “Y,” consisting of three equal-length arms (usually at a 120° angle). Each arm is approximately 30–40 cm long, 8–10 cm wide, and 15 cm high to prevent animals from escaping. The apparatus is placed in a disturbance-free laboratory environment, and a video tracking system is used to record animal behavior. Before the experiment, animals may be allowed to acclimate to the laboratory environment for about 30 min to reduce anxiety. The test begins by placing the animal in the start arm of the Y maze (typically a fixed arm), allowing it to explore freely for a set period (usually 5–10 min). During the experiment, the sequence and number of entries into each arm are recorded to calculate the alternation rate. A successful alternation is defined as consecutive entries into three different arms, such as A → B → C. If the animal revisits any arm within three consecutive choices, it is not counted as a successful alternation. The spontaneous alternation rate is calculated as: Spontaneous alternation rate = Number of successful alternations / Total exploration attempts.

### Nissl staining

Brain tissue from the mice was cut into 10 μm sections on a frozen slicer and pasted on a slide. The samples were fixed in 70% ethanol, then sequentially dehydrated in 100%, 90%, 80%, and 70% ethanol for 2 min each. After clearing with xylene, the sections were incubated in 1% tar purple (Solarbio, G1430) for 30 min. They were then rinsed with distilled water and differentiated in 70% alcohol for several minutes. Dehydration was repeated with 70%, 80%, and 95% ethanol for 2 min each, followed by 100% ethanol, before being sealed with neutral gum.

### Western blots

Mouse hippocampus protein lysates were resolved by sodium dodecyl sulfate-polyacrylamide gel electrophoresis (SDS-PAGE) and subsequently immunoblotted onto polyvinylidene difluoride (PVDF) membranes. Membranes were blocked with 5% nonfat milk at room temperature for 1 h. After TBST washing (three times, 5 min per wash), the membranes were incubated with the indicated primary antibodies at 4 °C overnight with shaking. The primary antibodies included: COX IV (Proteintech, 23274-1-AP), LaminB1 (Proteintech, 80906-1-RR), HIF-1α (Abcam, ab228649), STOML2 (Proteintech, 60052-1-AP), PGAM5 (Proteintech, 28445-1-AP), PINK1 (Abconal, A11435), LC3 (Sigma,L7543).After incubation, the membranes were washed three times and then incubated at room temperature for 1 h with secondary antibodies, including IRDye 680RD goat anti-mouse IgG (H + L) (Licor, 926-68070), IRDye 680RD goat anti-rabbit IgG (H + L) (Licor, 926-68071), IRDye 800CW goat anti-mouse IgG (H + L) (Licor, 926-32210), IRDye 800CW goat anti-rabbit IgG (H + L) (Licor, 926-32211). PVDF was scanned using a detection system (Odyssey, USA), and band intensities were normalized to Lamin B1 or COX IV. Statistical analyses were performed using ImageJ and GraphPad software.

### RT-PCR

An RNeasy kit (Qiagen, 74104) was used to extract total RNA from mice hippocampal tissue, and then the Transcriptor High Fidelity cDNA synthesis kit (Roche, 5081963001) was used to reverse transcribe the RNA into cDNA. All operations were according to the instructions. The following primers were used: Stoml2 for: TACAAGGCAAGTTACGGTGTGG; Stoml2 rev: GAGAATGCGCTGACATACTGCT; 18S sense: GTAACCCGTTGAACCCCATT; 18S anti: CCATCCAATCGGTAGTAGCG.

### Cytotoxicity detection

Cytotoxicity was detected by the LDH assay (Roche, 4744926001). The powder was dissolved in ddH2O and mixed thoroughly to make the catalytic solution. Then, 250 μL of the catalytic solution was added to the staining solution (11.25 mL) and mixed thoroughly. Then, 100 μL cell supernatant of each group was added to the new 96-well plate. The LDH reaction solution (100 μL) was added with subsequent incubation at room temperature away from light for 30 min. After the incubation, 50 μL stop solution was added to each well and gently mixed for 10 min. The OD value of each well was measured at 490 nm by a microplate reader.

### Cell Proliferation Assay

Cell proliferation was detected by the CCK8 assay, including the following steps: (1) Standard Curve: Count the cells in the suspension and prepare a cell concentration gradient. Dilute with culture medium to create 4-7 gradients, incubate overnight, then remove the medium and add fresh medium with CCK-8 reagent. After 1 h, measure OD to create a standard curve. (2) Cell Seeding: Seed cells at the optimal density determined, and incubate overnight. (3) Add CCK-8: Remove the original medium, and add 100 μl of medium with CCK-8. (4) Incubation: Incubate for 1 h, then transfer the hypoxia group to a 1% O2 incubator. (5) OD Measurement: Measure OD at 450 nm using a microplate reader.

### Cell death detection

PI/Hoechst detection was used to detect the cell death rate. Hoechst labels all cells with blue fluorescence, and PI labels only dead cells with red fluorescence. Therefore, the ratio of red to blue can be used to calculate the cell death rate. After the cells were treated, the original medium was discarded, and the cells were rinsed three times with PBS. The PI (Sigma, P4170) and Hoechst (Sigma, B2261) mixture was added into the cell culture well and incubated at 37 °C for 10 min under dark conditions. The cells were removed from the incubator, the mixture of PI and Hoechst was discarded, and the cells were rinsed three times with PBS. Confocal microscopy was used for observation and imaging.

### MitoTracker staining and flow cytometry detection

Using MitoTracker Green probe kit purchased from Thermo Corporation. (1) Prepare staining solution: dilute 1 mM MitoTracker stock in serum-free medium to a working concentration of 150 nM. (2) Staining: Once cells reach the desired density, discard the old medium, add pre-warmed MitoTracker solution, and incubate for 10 min in the dark. (3) Remove staining solution: Replace with regular medium. (4) Flow cytometry: Digest, centrifuge, and collect cells to create a single-cell suspension. Perform fluorescence detection using a flow cytometer with the FITC channel.

### Mitophagy inhibition

(1) Preparation of Mdivi-1 Working Solution: Dissolve Mdivi-1 (purchased from Selleck, S7162) in DMSO to prepare a stock solution and store it at −20 °C in the dark. Before use, dilute the stock solution in complete culture medium to a final concentration of 10 µM. (2) Cell Culture: Seed cells in a 96-well plate and incubate at 37 °C with 5% CO₂ until they reach the appropriate density. (3) Cell Treatment: Remove the original culture medium and replace it with fresh medium containing Mdivi-1 at a ratio of 10 µl per 1 ml of medium. Pre-treat the cells for 30 min. (4) Subsequent Assays: Perform cell viability and functional assays such as CCK-8.

### Statistical analysis

Excel and GraphPad Prism 9.0 software were used for data preservation, recording, statistics, and analyses. Image data were analyzed by ImageJ and other software. All results were analyzed using the *t* test, one-way ANOVA, and two-way ANOVA as appropriate. Data are expressed as the mean ± standard error (mean ± SEM), with *P* ≤ 0.05 as a significant difference. In animal behavioral tests, *n* ≥ 10; in protein detection experiments, such as Western blot and immunofluorescence, *n* ≥ 3.

## Supplementary information

Supplementary figure

Full uncropped Gels and Blots image

## Data Availability

The datasets generated and analyzed during the current study are available from the corresponding author on reasonable request.

## References

[1] Li S, Hafeez A, Noorulla F, Geng X, Shao G, Ren C, et al. Preconditioning in neuroprotection: from hypoxia to ischemia. Prog Neurobiol. 2017;157:79–91. 28110083 10.1016/j.pneurobio.2017.01.001 PMC5515698

[2] Li B, Yang WW, Yao BC, Chen QL, Zhao LL, Song YQ, et al. Liriodendrin alleviates myocardial ischemia‑reperfusion injury via partially attenuating apoptosis, inflammation and mitochondria damage in rats. Int J Mol Med. 2025;55:1–12. 39981888 10.3892/ijmm.2025.5506 PMC11875722

[3] Alshial EE, Abdulghaney MI, Wadan AS, Abdellatif MA, Ramadan NE, Suleiman AM, et al. Mitochondrial dysfunction and neurological disorders: A narrative review and treatment overview. Life Sci. 2023;334:122257. 37949207 10.1016/j.lfs.2023.122257

[4] Schmitt, LO & Gaspar, JM obesity-induced brain neuroinflammatory and mitochondrial changes. Metabolites. 2023;13. 10.3390/metabo13010086 10.3390/metabo13010086 PMC9865135 36677011

[5] Hoffmann L, Waclawczyk MS, Tang S, Hanschmann EM, Gellert M, Rust MB, et al. Cofilin1 oxidation links oxidative distress to mitochondrial demise and neuronal cell death. Cell Death Dis. 2021;12:953. 34657120 10.1038/s41419-021-04242-1 PMC8520533

[6] Liang R, Hou X, Zhou D, Zhu L, Teng L, Song W, et al. Exercise preconditioning mitigates Ischemia-Reperfusion injury in rats by enhancing mitochondrial respiration. Neuroscience. 2024;562:64–74. 39461659 10.1016/j.neuroscience.2024.10.045

[7] Wen P, Sun Z, Gou F, Wang J, Fan Q, Zhao D, et al. Oxidative stress and mitochondrial impairment: Key drivers in neurodegenerative disorders. Ageing Res Rev. 2025;104:102667. 39848408 10.1016/j.arr.2025.102667

[8] Almeida VN. Somatostatin and the pathophysiology of Alzheimer’s disease. Ageing Res Rev. 2024;96:102270. 38484981 10.1016/j.arr.2024.102270

[9] Szczepanowska K, Trifunovic A. Mitochondrial matrix proteases: quality control and beyond. FEBS J. 2022;289:7128–46. 33971087 10.1111/febs.15964

[10] Tian RZ, Zhuang DL, Vong CT, He X, Ouyang Q, Liang JH, et al. Role of Autophagy in Myocardial Remodeling After Myocardial Infarction. J Cardiovasc Pharm. 2025;85:1–11. 10.1097/FJC.0000000000001646 39454200

[11] Cheng Y, Gu W, Wu X, Tian W, Mu Z, Ye Y, et al. Allicin alleviates traumatic brain injury-induced neuroinflammation by enhancing PKC-δ-mediated mitophagy. Phytomedicine. 2025;139:156500. 39986225 10.1016/j.phymed.2025.156500

[12] Meng Q, Mi Y, Xu L, Liu Y, Liang D, Wang Y, et al. A quinolinyl analog of resveratrol improves neuronal damage after ischemic stroke by promoting Parkin-mediated mitophagy. Chin J Nat Med. 2025;23:214–24. 39986697 10.1016/S1875-5364(25)60825-9

[13] Wu H, Chen Q. Hypoxia activation of mitophagy and its role in disease pathogenesis. Antioxid Redox Signal. 2015;22:1032–46. 25526784 10.1089/ars.2014.6204

[14] Liu L, Feng D, Chen G, Chen M, Zheng Q, Song P, et al. Mitochondrial outer-membrane protein FUNDC1 mediates hypoxia-induced mitophagy in mammalian cells. Nat Cell Biol. 2012;14:177–85. 22267086 10.1038/ncb2422

[15] Kuang Y, Ma K, Zhou C, Ding P, Zhu Y, Chen Q, et al. Structural basis for the phosphorylation of FUNDC1 LIR as a molecular switch of mitophagy. Autophagy. 2016;12:2363–73. 27653272 10.1080/15548627.2016.1238552 PMC5173264

[16] Ge P, Dawson VL, Dawson TM. PINK1 and Parkin mitochondrial quality control: a source of regional vulnerability in Parkinson’s disease. Mol Neurodegener. 2020;15:20. 32169097 10.1186/s13024-020-00367-7 PMC7071653

[17] Linqing L, Yuhan Q, Erfei L, Yong Q, Dong W, Chengchun T, et al. Hypoxia-induced PINK1/Parkin-mediated mitophagy promotes pulmonary vascular remodeling. Biochem Biophys Res Commun. 2021;534:568–75. 33239167 10.1016/j.bbrc.2020.11.040

[18] Shao Q, Liu J, Li G, Gu Y, Guo M, Guan Y, et al. Proteomic analysis reveals that mitochondria dominate the hippocampal hypoxic response in mice. Int J Mol Sci. 2022;23:14094. 36430571 10.3390/ijms232214094 PMC9697535

[19] Cheng M, Lin N, Dong D, Ma J, Su J, Sun L. PGAM5: A crucial role in mitochondrial dynamics and programmed cell death. Eur J Cell Biol. 2021;100:151144. 33370650 10.1016/j.ejcb.2020.151144

[20] Zeb A, Choubey V, Gupta R, Kuum M, Safiulina D, Vaarmann A, et al. A novel role of KEAP1/PGAM5 complex: ROS sensor for inducing mitophagy. Redox Biol. 2021;48:102186. 34801863 10.1016/j.redox.2021.102186 PMC8607199

[21] Yan C, Gong L, Chen L, Xu M, Abou-Hamdan H, Tang M, et al. PHB2 (prohibitin 2) promotes PINK1-PRKN/Parkin-dependent mitophagy by the PARL-PGAM5-PINK1 axis. Autophagy. 2020;16:419–34. 31177901 10.1080/15548627.2019.1628520 PMC6999623

[22] Fan R, Jiang H, Hu Y, Xu Y, Zhou Y, Chen G, et al. Stomatin-like protein-2 attenuates macrophage pyroptosis and H9c2 cells apoptosis by protecting mitochondrial function. Biochem Biophys Res Commun. 2022;636:112–20. 36332472 10.1016/j.bbrc.2022.10.047

[23] Christie DA, Mitsopoulos P, Blagih J, Dunn SD, St-Pierre J, Jones RG, et al. Stomatin-like protein 2 deficiency in T cells is associated with altered mitochondrial respiration and defective CD4+ T cell responses. J Immunol. 2012;189:4349–60. 23028053 10.4049/jimmunol.1103829

[24] Guo H, Liang S, Wang Y, Zhou S, Yin D, Zhang S, et al. Cytochrome B5 type A alleviates HCC metastasis via regulating STOML2 related autophagy and promoting sensitivity to ruxolitinib. Cell Death Dis. 2022;13:623. 35851063 10.1038/s41419-022-05053-8 PMC9293983

[25] Ma W, Chen Y, Xiong W, Li W, Xu Z, Wang Y, et al. STOML2 interacts with PHB through activating MAPK signaling pathway to promote colorectal Cancer proliferation. J Exp Clin Cancer Res. 2021;40:359. 34781982 10.1186/s13046-021-02116-0 PMC8591804

[26] Wai T, Saita S, Nolte H, Müller S, König T, Richter-Dennerlein R, et al. The membrane scaffold SLP2 anchors a proteolytic hub in mitochondria containing PARL and the i-AAA protease YME1L. EMBO Rep. 2016;17:1844–56. 27737933 10.15252/embr.201642698 PMC5283581

[27] Tang T, Hu LB, Ding C, Zhang Z, Wang N, Wang T, et al. Src inhibition rescues FUNDC1-mediated neuronal mitophagy in ischaemic stroke. Stroke Vasc Neurol. 2024;9:367–79. 37793899 10.1136/svn-2023-002606 PMC11420917

[28] Chen G, Cizeau J, Vande Velde C, Park JH, Bozek G, Bolton J, et al. Nix and Nip3 form a subfamily of pro-apoptotic mitochondrial proteins. J Biol Chem. 1999;274:7–10. 9867803 10.1074/jbc.274.1.7

[29] Hu S, Zhang C, Ni L, Huang C, Chen D, Shi K, et al. Stabilization of HIF-1α alleviates osteoarthritis via enhancing mitophagy. Cell Death Dis. 2020;11:481. 32587244 10.1038/s41419-020-2680-0 PMC7316774

[30] Randle RK, Amara VR, Popik W. IFI16 is indispensable for promoting HIF-1α-mediated APOL1 expression in human podocytes under hypoxic conditions. Int J Mol Sci. 2024;25:3324. 38542298 10.3390/ijms25063324 PMC10970439

[31] Jewell UR, Kvietikova I, Scheid A, Bauer C, Wenger RH, Gassmann M. Induction of HIF-1alpha in response to hypoxia is instantaneous. FASEB J. 2001;15:1312–4. 11344124

[32] Liu Y, Liu WC, Sun Y, Shen X, Wang X, Shu H, et al. Normobaric hyperoxia extends neuro- and vaso-protection of N-acetylcysteine in transient focal ischemia. Mol Neurobiol. 2017;54:3418–27. 27177548 10.1007/s12035-016-9932-0

[33] Zhang Z, Yan J, Shi H. Role of hypoxia inducible factor 1 in hyperglycemia-exacerbated blood-brain barrier disruption in ischemic stroke. Neurobiol Dis. 2016;95:82–92. 27425889 10.1016/j.nbd.2016.07.012 PMC5010995

[34] Zheng Y, Huang C, Lu L, Yu K, Zhao J, Chen M, et al. STOML2 potentiates metastasis of hepatocellular carcinoma by promoting PINK1-mediated mitophagy and regulates sensitivity to lenvatinib. J Hematol Oncol. 2021;14:16. 33446239 10.1186/s13045-020-01029-3 PMC7807703

[35] Lazarou M, Sliter DA, Kane LA, Sarraf SA, Wang C, Burman JL, et al. The ubiquitin kinase PINK1 recruits autophagy receptors to induce mitophagy. Nature. 2015;524:309–14. 26266977 10.1038/nature14893 PMC5018156

[36] Pedrera L, Prieto Clemente L, Dahlhaus A, Lotfipour Nasudivar S, Tishina S, Olmo González D, et al. Ferroptosis triggers mitochondrial fragmentation via Drp1 activation. Cell Death Dis. 2025;16:40. 39863602 10.1038/s41419-024-07312-2 PMC11762985

[37] Qin X, Wang R, Xu H, Tu L, Chen H, Li H, et al. Identification of an autoinhibitory, mitophagy-inducing peptide derived from the transmembrane domain of USP30. Autophagy. 2022;18:2178–97. 34989313 10.1080/15548627.2021.2022360 PMC9397470

[38] Ling Z, Ge X, Jin C, Song Z, Zhang H, Fu Y, et al. Copper doped bioactive glass promotes matrix vesicles-mediated biomineralization via osteoblast mitophagy and mitochondrial dynamics during bone regeneration. Bioact Mater. 2025;46:195–212. 39760064 10.1016/j.bioactmat.2024.12.010 PMC11699476

[39] Li K, Xia X, Tong Y. Multiple roles of mitochondrial autophagy receptor FUNDC1 in mitochondrial events and kidney disease. Front Cell Dev Biol. 2024;12:1453365. 39445333 10.3389/fcell.2024.1453365 PMC11496291

[40] Martinez A, Sanchez-Martinez A, Pickering JT, Twyning MJ, Terriente-Felix A, Chen PL, et al. Mitochondrial CISD1/Cisd accumulation blocks mitophagy and genetic or pharmacological inhibition rescues neurodegenerative phenotypes in Pink1/parkin models. Mol Neurodegeneration. 2024;19:12. 10.1186/s13024-024-00701-3 PMC10811860 38273330

[41] Yi J, Wang HL, Lu G, Zhang H, Wang L, Li ZY, et al. Spautin-1 promotes PINK1-PRKN-dependent mitophagy and improves associative learning capability in an alzheimer disease animal model. Autophagy. 2024;20:2655–76. 39051473 10.1080/15548627.2024.2383145 PMC11587853

[42] Eldeeb MA, Fallahi A, Soumbasis A, Bayne AN, Trempe JF, Fon EA. Mitochondrial import stress and PINK1-mediated mitophagy: the role of the PINK1-TOMM-TIMM23 supercomplex. Autophagy. 2024;20:1903–5. 38597070 10.1080/15548627.2024.2340399 PMC11262206

[43] Shin HS, Park GH, Choi ES, Park SY, Kim DS, Chang J, et al. RNF213 variant and autophagic impairment: a pivotal link to endothelial dysfunction in moyamoya disease. J Cereb Blood Flow Metab : Off J Int Soc Cereb Blood Flow Metab. 2024;44:1801–15. 10.1177/0271678X241245557 PMC11494856 38573771

[44] Lan XY, Li D, Cui Y, Nguyen TN, Li S, Chen HS. Proteomic analysis of jugular venous blood in acute large vessel occlusion stroke with futile recanalization. J Cereb Blood Flow Metab. 2024;44:702–11. 38000017 10.1177/0271678X231216767 PMC11197136

[45] Zhao YH, Liang Y, Wang KJ, Jin SN, Yu XM, Zhang Q, et al. Endothelial lincRNA-p21 alleviates cerebral ischemia/reperfusion injury by maintaining blood-brain barrier integrity. J Cereb Blood Flow Metab. 2024;44:1532–50. 38661094 10.1177/0271678X241248907 PMC11418693

[46] Wong Zhang DE, Gibson Hughes TA, Figueiredo Galvao HB, Lo C, Dinh QN, Zhang SR, et al. Post-stroke cognitive impairment and brain hemorrhage are augmented in hypertensive mice. J Cereb Blood Flow Metab. 2024;44:1517–34. 38886874 10.1177/0271678X241262127 PMC11572097

[47] Zhou K, Tan Y, Zhang G, Li J, Xing S, Chen X, et al. Loss of SARM1 ameliorates secondary thalamic neurodegeneration after cerebral infarction. J Cereb Blood Flow Metab. 2024;44:224–38. 37898107 10.1177/0271678X231210694 PMC10993876

[48] Gu R, Bai L, Yan F, Zhang S, Zhang X, Deng R, et al. Thioredoxin-1 decreases alpha-synuclein induced by MPTP through promoting autophagy-lysosome pathway. Cell Death Discov. 2024;10:93. 38388451 10.1038/s41420-024-01848-0 PMC10884002

[49] Park J, Jin L, Song HC, Chen Y, Jang EY, Park GH, et al. CO confers neuroprotection via activating the PERK-calcineurin pathway and inhibiting necroptosis. Cell Death Discov. 2025;11:254. 40425550 10.1038/s41420-025-02530-9 PMC12116729

[50] Su Y, Ke C, Li C, Huang C, Wan C. Intermittent hypoxia promotes the recovery of motor function in rats with cerebral ischemia by regulating mitochondrial function. Exp Biol Med (Maywood). 2022;247:1364–78. 35665627 10.1177/15353702221098962 PMC9442452

[51] Peng W, Ma H, Zhao R, Xu S, Lv M, Jing B, et al. Role of intermittent hypoxic training combined with methazolamide in the prevention of high-altitude cerebral edema in rats. Sci Rep. 2024;14:30252. 39632926 10.1038/s41598-024-81226-z PMC11618614

[52] Wang X, Gong L, Wei C, Zhao Y, Ran L, Li P, et al. Inhibition of NSUN6 protects against intermittent hypoxia-induced oxidative stress and inflammatory response in adipose tissue through suppressing macrophage ferroptosis and M1 polarization. Life Sci. 2025;364:123433. 39884342 10.1016/j.lfs.2025.123433

[53] Panerai RB, Davies A, Clough RH, Beishon LC, Robinson TG, Minhas JS. The effect of hypercapnia on the directional sensitivity of dynamic cerebral autoregulation and the influence of age and sex. J Cereb Blood Flow Metab. 2024;44:272–83. 37747437 10.1177/0271678X231203475 PMC10993882

[54] Sawan H, Li C, Buch S, Bernitsas E, Haacke EM, Ge Y, et al. Reduced oxygen extraction fraction in deep cerebral veins associated with cognitive impairment in multiple sclerosis. J Cereb Blood Flow Metab. 2024;44:1298–305. 38820447 10.1177/0271678X241259551 PMC11342723

[55] Cheng X, Potenza DM, Brenna A, Ajalbert G, Yang Z, Ming XF. Aging increases hypoxia-induced endothelial permeability and blood-brain barrier dysfunction by upregulating arginase-II. Aging Dis. 2024;15:2710–5415. 38300641 10.14336/AD.2023.1225 PMC11567255

[56] Kataura T, Wilson N, Ma G, Korolchuk VI. Mitophagy as a guardian against cellular aging. Autophagy. 2025;21:249–51. 39402011 10.1080/15548627.2024.2414461 PMC11702949

