# Supplementary material for: Novel mechanism of neuronal hypoxia response: HIF-1α/STOML2 mediated PINK1-dependent mitophagy activation against neuronal injury
Source: Cell Death Discov. 2026 Feb 21;12:104. doi: 10.1038/s41420-026-02960-z (PMC12949251; doi:10.1038/s41420-026-02960-z)
Supplement: Supplementary file 1 — Supplementary figure [file 41420_2026_2960_MOESM1_ESM.docx]

**
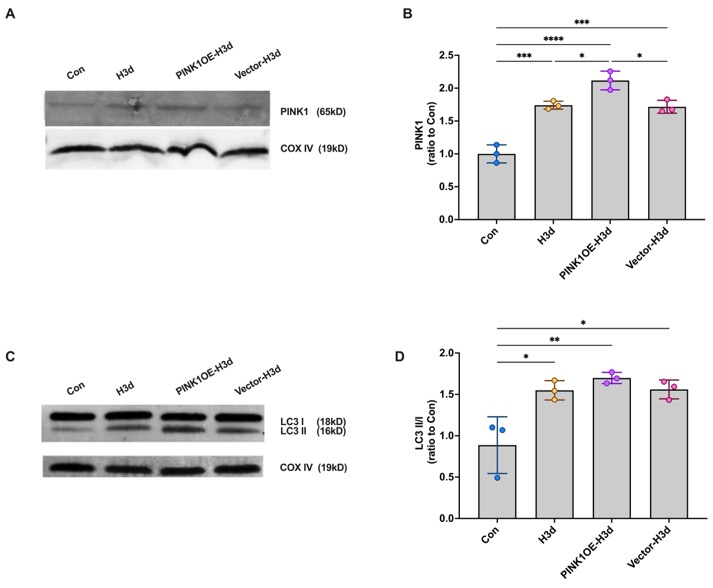
**

**Figure S1. PINK1 overexpression enhances the level of mitophagy.** **(A)** The levels of PINK1 in mice of each group in the hippocampus mitochondrial protein were detected by western blotting using COX IV as the internal reference. **(B)** Statistical analysis of PINK1 in mice of each group. **(C)** The levels of LC3 in the hippocampus mitochondrial protein were detected by western blotting using COX IV as the internal reference. **(D)** Statistical analysis of LC3 II/I in mice of each group. Data was analyzed via one-way ANOVA and Tukey’s post-test. *p < 0.05, **p < 0.01, ***p < 0.001, ****p < 0.0001.


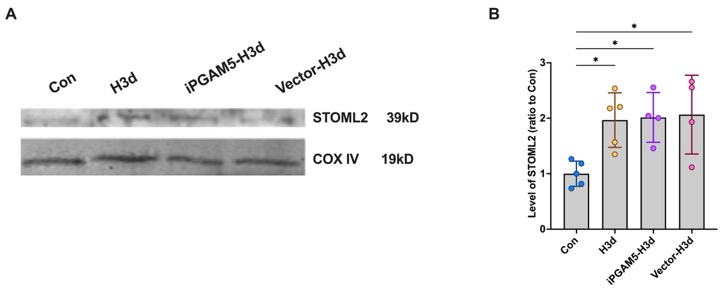


**Figure S2. STOML2 acts as an upstream regulator of PGAM5.** **(A)** The levels of STOML2 in mice of each group in the hippocampus mitochondrial protein were detected by western blotting using COX IV as the internal reference. **(B)** Statistical analysis of PINK1 in mice of each group. Data was analyzed via one-way ANOVA and Tukey’s post-test. *p < 0.05.
