# Supplementary material for: Novel mechanism of neuronal hypoxia response: HIF-1α/STOML2 mediated PINK1-dependent mitophagy activation against neuronal injury
Source: Cell Death Discov. 2026 Feb 21;12:104. doi: 10.1038/s41420-026-02960-z (PMC12949251; doi:10.1038/s41420-026-02960-z)
Supplement: Supplementary file 2 — Full uncropped Gels and Blots image [file 41420_2026_2960_MOESM2_ESM.docx]

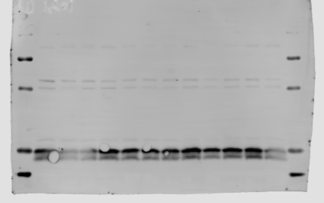
Full uncropped Gels and Blots image(s) for Figure1F


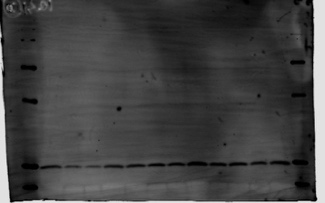

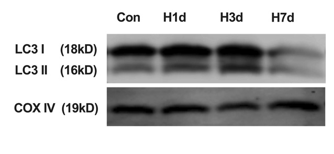


**COX IV**

**Con H1d H3d H7d**

**Con H1d H3d H7d**

**LC3 I**

**LC3II**

**10kD**

**15kD**

**35kD**

**55kD**

**10kD**

**55kD**

**35kD**

**15kD**

Full uncropped Gels and Blots image(s) for Figure1N


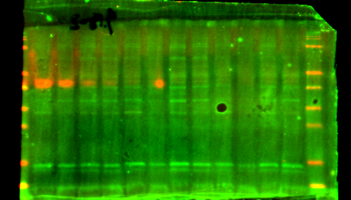

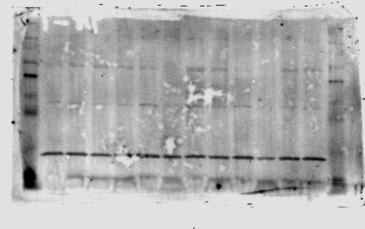

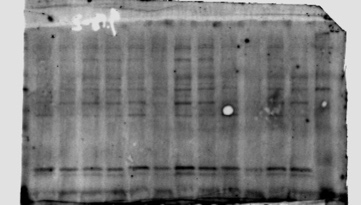

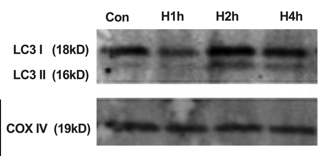


**10kD**

**15kD**

**55kD**

**25kD**

**LC3 I**

**LC3II**

**Con H1h H2h H4h**

**55kD**

**15kD**

**10kD**

**35kD**

**25kD**

**35kD**

**COX IV**

**Con H1h H2h H4h**

Full uncropped Gels and Blots image(s) for Figure2A


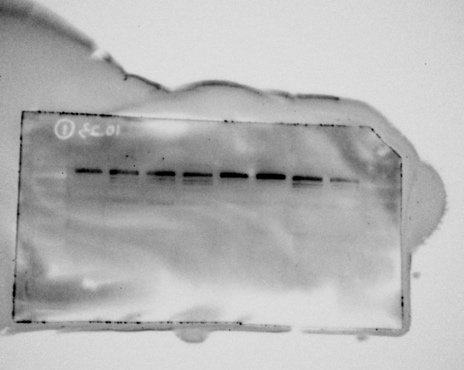

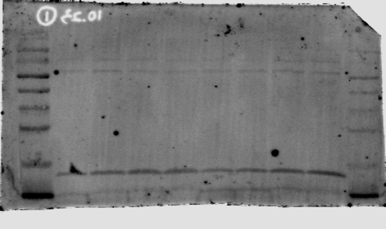

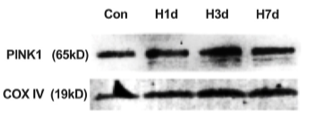


**COX IV**

**PINK1**

**Con H1d H3d H7d**

**Con H1d H3d H7d**

**70kD**

**50kD**

**40kD**

**35kD**

**25kD**

**20kD**

**15kD**

**10kD**

Full uncropped Gels and Blots image(s) for Figure2C


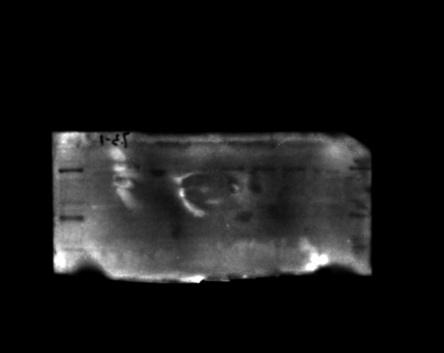

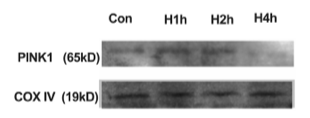


**10kD**

**PINK1**

**Con H1h H2h H4h**

**1****0kD**

**15kD**

**35kD**

**55kD**

**70kD**


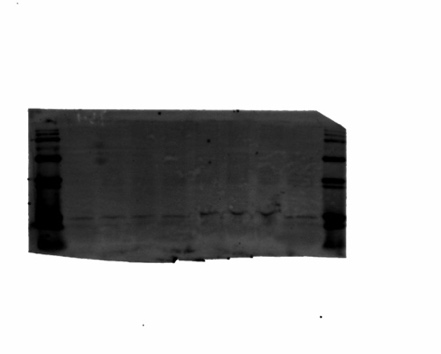


**70kD**

**Con H1h H2h H4h**

**15kD**

**55kD**

**35kD**

**COX IV**

**10kD**

Full uncropped Gels and Blots image(s) for Figure2K


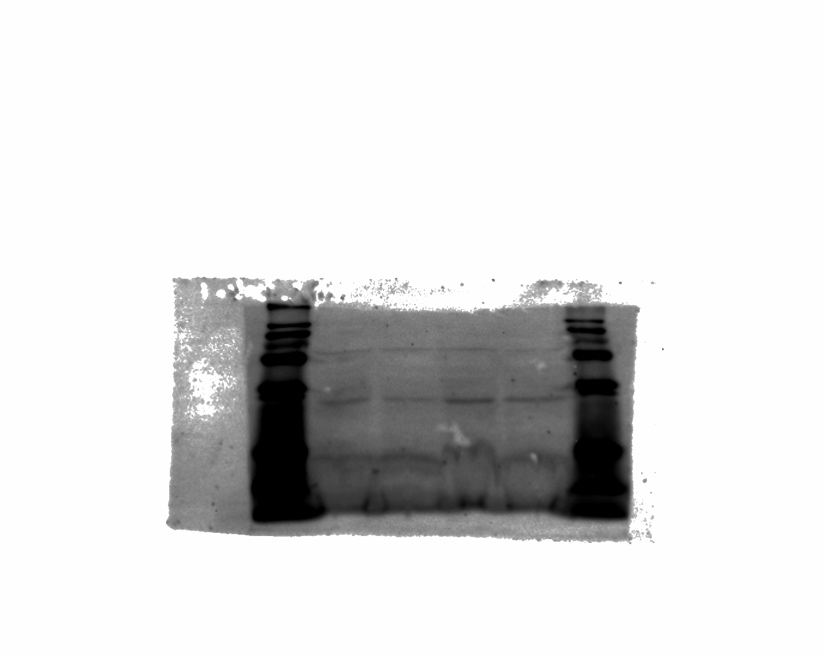

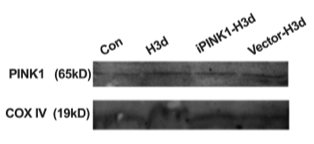


**PINK1**

**COX IV**

**Con H3d iPINK1-H3d Vestor-H3d**

**15kD**

**70kD**

**55kD**

**35kD**

Full uncropped Gels and Blots image(s) for Figure2M


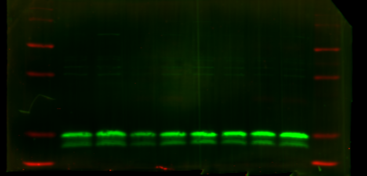

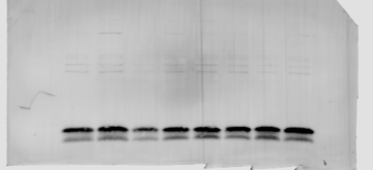

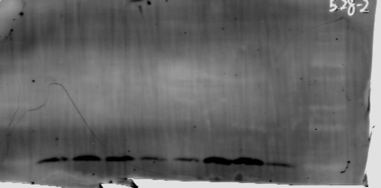

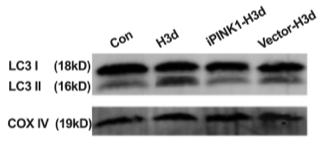


**COX IV**

**Con**

**H3d**

**Vestor-H3d**

**iPINK1-H3d**

**35kD**

**25kD**

**15kD**

**10kD**

**35kD**

**Vestor-H3d**

**iPINK1-H3d**

**H3d**

**Con**

**LC3 I**

**LC3 II**

**10kD**

**15kD**

Full uncropped Gels and Blots image(s) for Figure3A


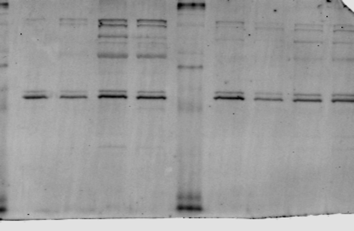

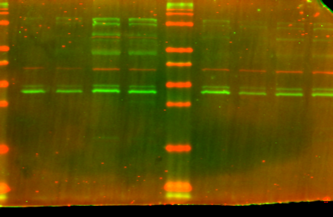


**Con H1d H3d. H7d**

**L-PGAM5**

**S-PGAM5**

**55kD**

**25kD**

**35kD**

**15kD**

**10kD**


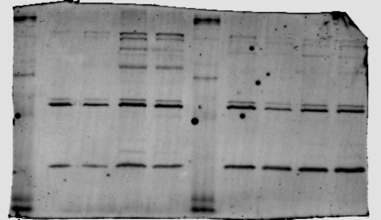

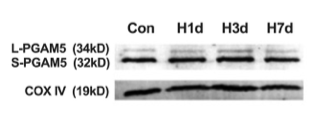


**Con H1d H3d. H7d**

**COX IV**

Full uncropped Gels and Blots image(s) for Figure3C


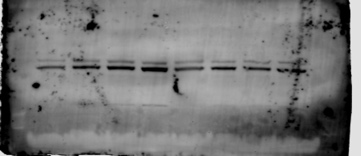

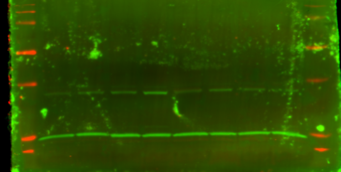

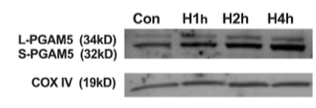


**L-PGAM5**

**S-PGAM5**

**Con H1h H2h. H4h**


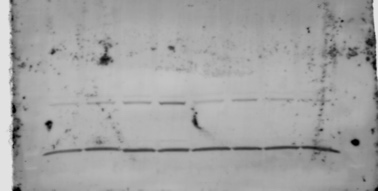


**Con H1h H2h. H4h**

**COX IV**

Full uncropped Gels and Blots image(s) for Figure3K


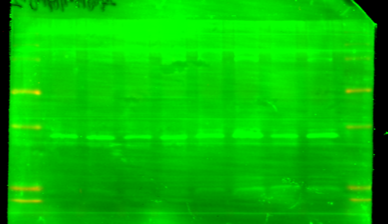

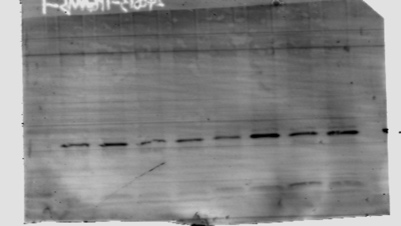

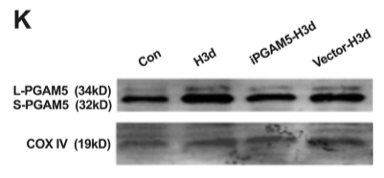


**L-PGAM5**

**S-PGAM5**

**COX IV**

**Con H3d iPGAM5-H3d Vestor-H3d**

**55kD**

**35kD**

**15kD**

**10kD**

Full uncropped Gels and Blots image(s) for Figure3M


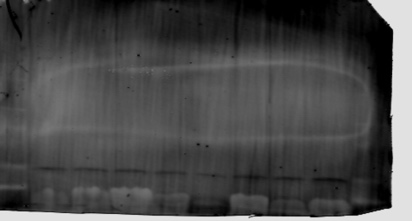

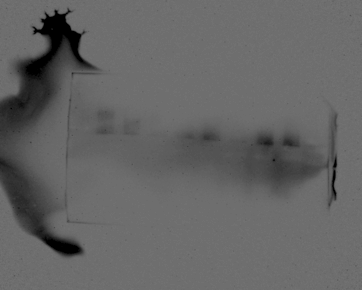

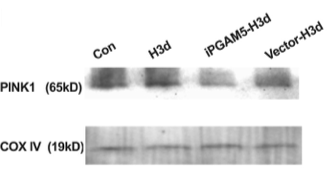


**PINK1**

**COX IV**

**15kD**

**35kD**

**55kD**

**70kD**

**Con**

**H3d**

**iPGAM5-H3d**

**Vestor-H3d**

**Vestor-H3d**

**iPGAM5-H3d**

**H3d**

**Con**

**10kD**

Full uncropped Gels and Blots image(s) for Figure3O


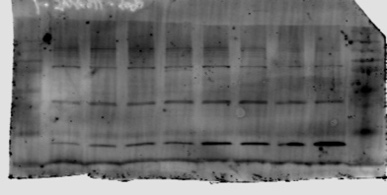

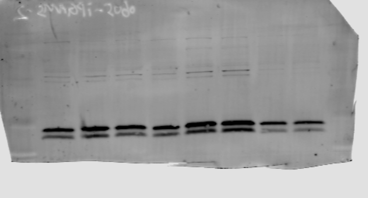

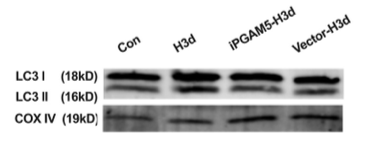


**COX IV**

**LC3 I**

**LC3 II**

**H3d**

**Con**

**Con**

**H3d**

**Vestor-H3d**

**iPGAM5-H3d**

**iPGAM5-H3d**

**Vestor-H3d**

**15kD**

**55kD**

**70kD**

**35kD**

**10kD**

Full uncropped Gels and Blots image(s) for Figure4A


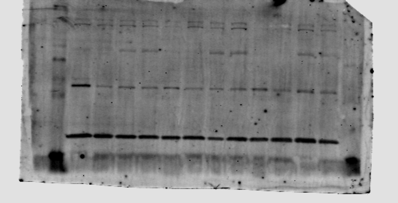

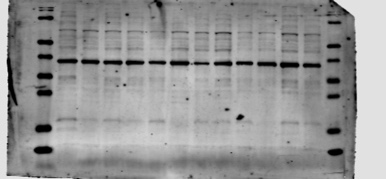

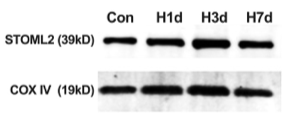


**COX IV**

**STOML2**

**Con H1d H3d H7d**

**Con H1d H3d H7d**

**25kD**

**35kD**

**55kD**

**15kD**

**70kD**

**10kD**

Full uncropped Gels and Blots image(s) for Figure4C


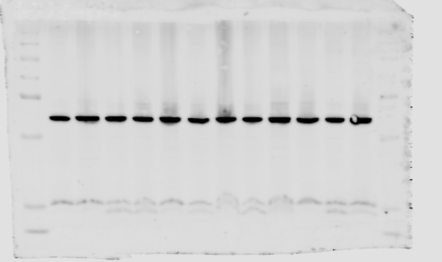

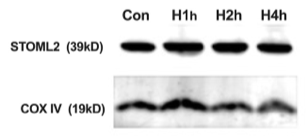


**STOML2**

**COX IV**

**Con H1h H2h H4h**

**55kD**

**35kD**

**10kD**

**15kD**

Full uncropped Gels and Blots image(s) for Figure4K


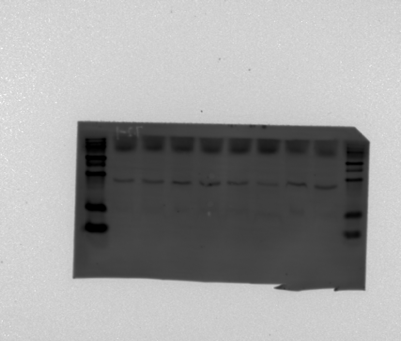

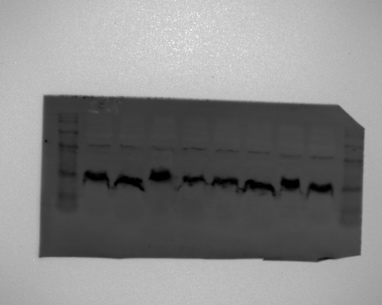

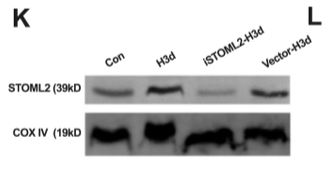


**STOML2**

**COX IV**

**Con**

**H3d**

**iSTOML2-H3d**

**Vestor-H3d**

**H3d**

**Con**

**iSTOML2-H3d**

**Vestor-H3d**

**35kD**

**15kD**

**10kD**

**55kD**

Full uncropped Gels and Blots image(s) for Figure4M


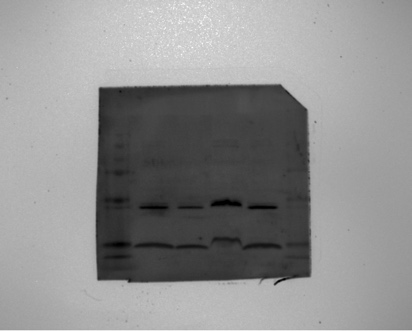

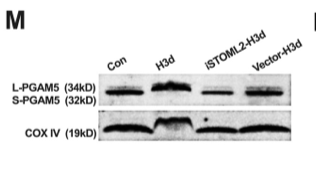


**35kD**

**55kD**

**15kD**

**Con**

**H3d**

**Vestor-H3d**

**iSTOML2-H3d**

**L-PGAM5**

**S-PGAM5**

**COX IV**

**10kD**

Full uncropped Gels and Blots image(s) for Figure4O


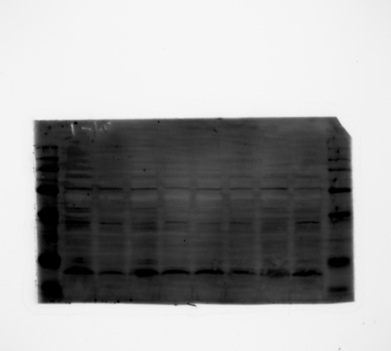

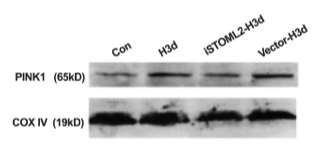


**PINK1**

**COX IV**

**iSTOML2-H3d**

**Vestor-H3d**

**Con**

**H3d**

**25kD**

**35kD**

**10kD**

**15kD**

**55kD**

**70kD**

Full uncropped Gels and Blots image(s) for Figure4Q


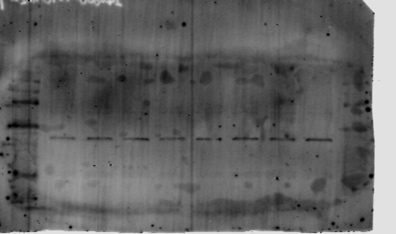

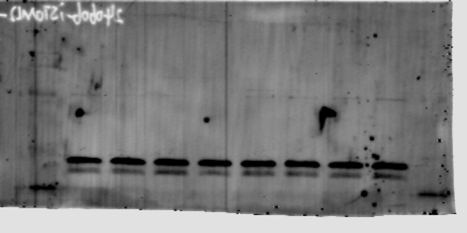

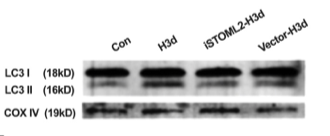


**COX IV**

**LC3 I**

**LC3 II**

**iSTOML2-H3d**

**Vestor-H3d**

**Con**

**H3d**

**H3d**

**Con**

**Vestor-H3d**

**iSTOML2-H3d**

**35kD**

**15kD**

**10kD**

**55kD**

**15kD**

**10kD**

Full uncropped Gels and Blots image(s) for Figure5A


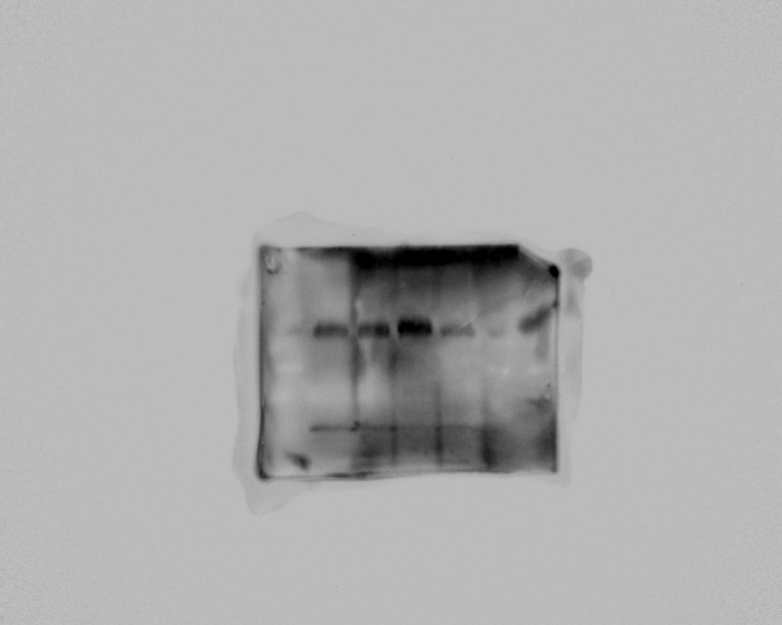

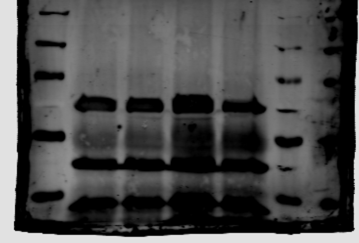

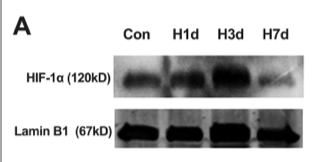


**55kD**

**150kD**

**100kD**

**70kD**

**Lamin B1**

**HIF-1α**

**Con H1d H3d H7d**

**Con H1d H3d H7d**

Full uncropped Gels and Blots image(s) for Figure5C


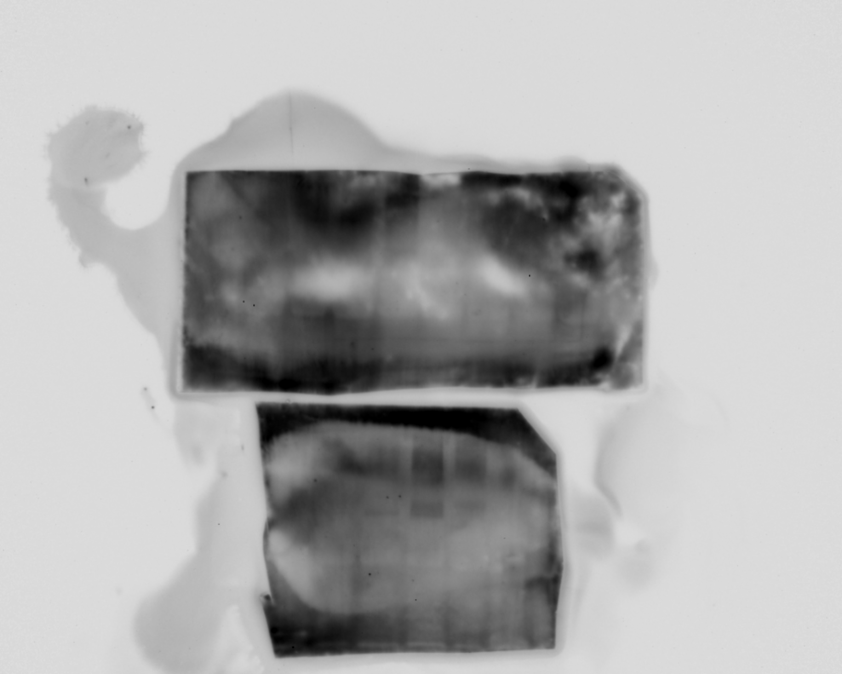

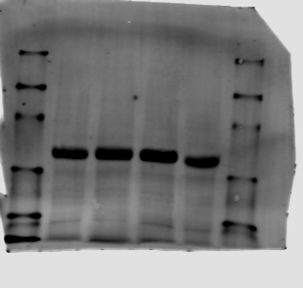

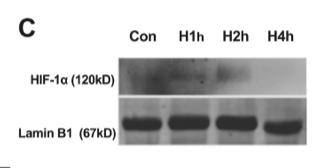


**HIF-1α**

**Lamin B1**

**Con H1h H2h H4h**

**Con H1h H2h H4h**

**70kD**

**100kD**

**150kD**

**55kD**

Full uncropped Gels and Blots image(s) for Figure6D


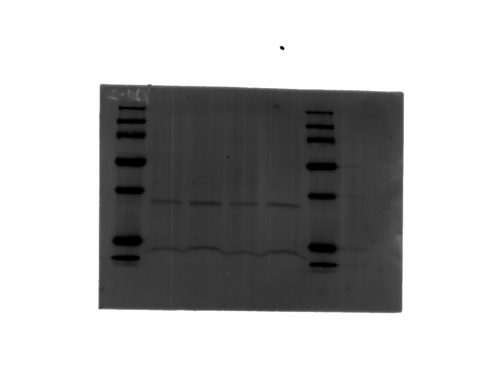


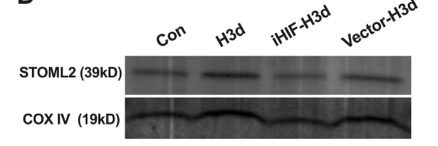


**15kD**

**35kD**

**55kD**

**10kD**

**COX IV**

**STOML2**

Full uncropped Gels and Blots image(s) for Figure6F


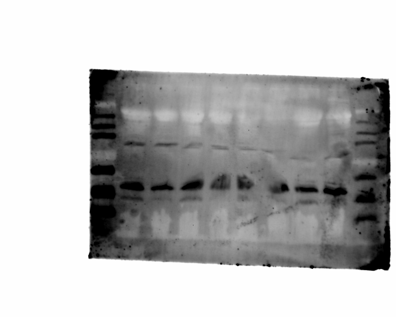


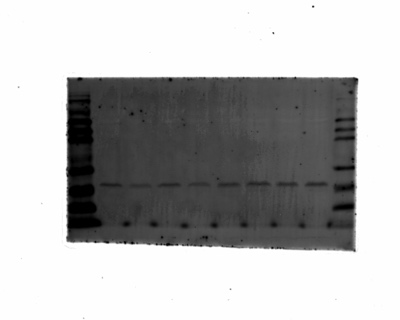

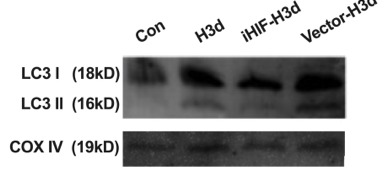


**Con**

**H3d**

**Vestor-H3d**

**iHIF-H3d**

**10kD**

**15kD**

**25kD**

**LC3 I**

**LC3 II**

**Vestor-H3d**

**iHIF-H3d**

**Con**

**H3d**

**25kD**

**15kD**

**COX IV**

**10kD**

Full uncropped Gels and Blots image(s) for Figure7A


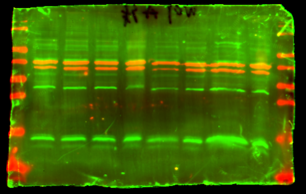

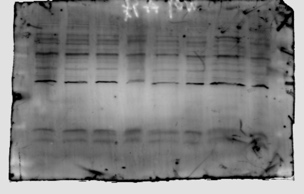

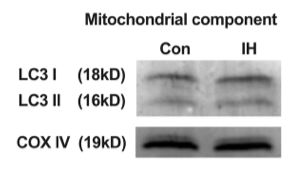


**LC3 I**

**LC3 II**

**Con. IH**

**55kD**

**35kD**

**10kD**

**15kD**

**25kD**


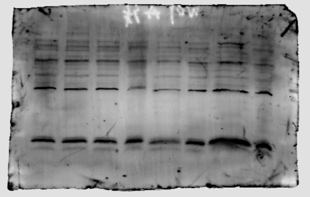


**COX IV**

**Con. IH**

Full uncropped Gels and Blots image(s) for Figure7C


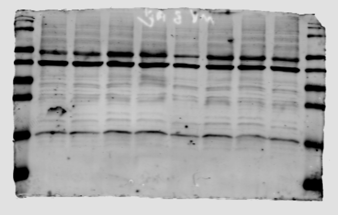

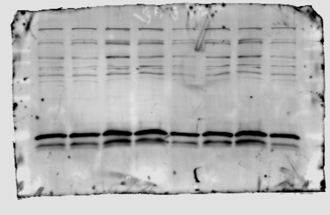

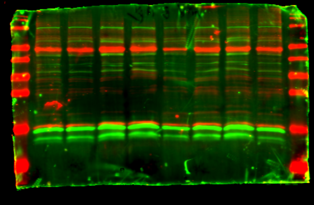

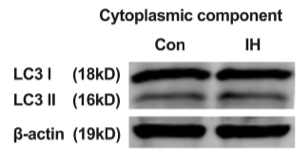


**35kD**

**Con IH**

**25kD**

**55kD**

**15kD**

**10kD**

**Con IH**

**β-actin**

**COX IV**

Full uncropped Gels and Blots image(s) for Figure7E


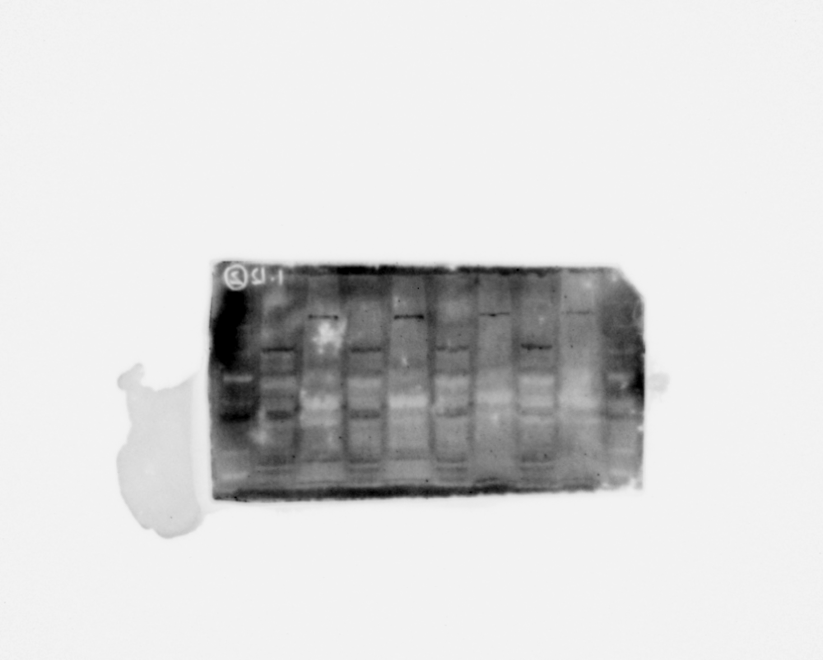

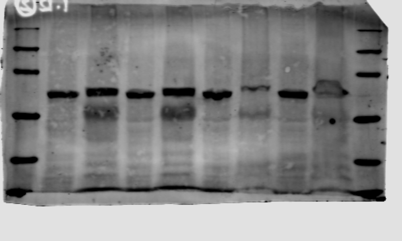

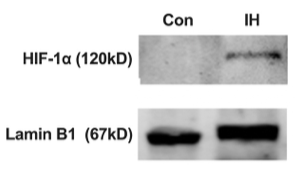


**HIF-1α**

**Con IH**

**70kD**

**100kD**

**150kD**

**150kD**

**100kD**

**Con IH**

**70kD**

**Lamin B1**

**55kD**

Full uncropped Gels and Blots image(s) for Figure7H


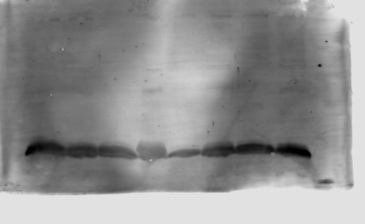

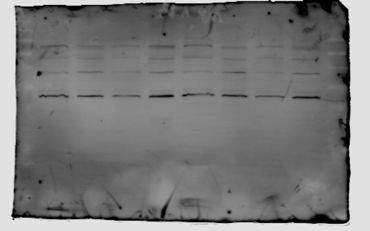

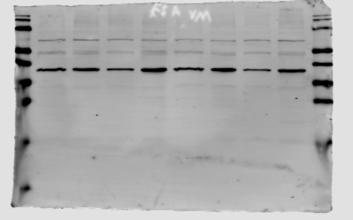

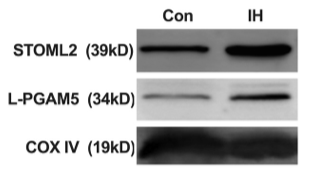


**L-PGAM5**

**S-PGAM5**

**STOML2**

**70kD**

**55kD**

**25kD**

**35kD**

**15kD**

**10kD**

**Con IH**

**Con IH**


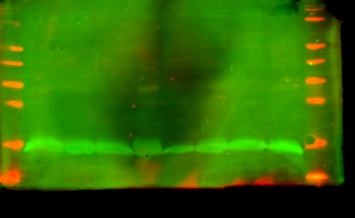


**COX IV**

**Con IH**

Full uncropped Gels and Blots image(s) for Figure7K


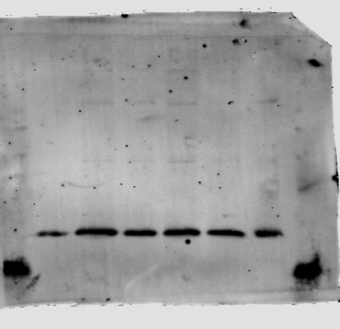

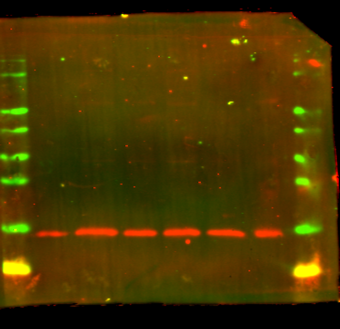


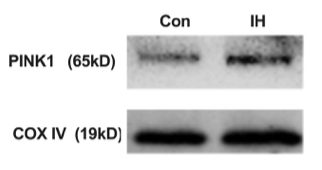


**55kD**

**70kD**

**35kD**

**25kD**

**15kD**

**10kD**

**Con IH**

**Con IH**

**PINK1**

**COX IV**
